# High expression of B7-H3 on stromal cells defines tumor and stromal compartments in epithelial ovarian cancer and is associated with limited immune activation

**DOI:** 10.1186/s40425-019-0816-5

**Published:** 2019-12-31

**Authors:** Heather L. MacGregor, Azin Sayad, Andrew Elia, Ben X. Wang, Sarah Rachel Katz, Patricia A. Shaw, Blaise A. Clarke, Sarah Q. Crome, Celine Robert-Tissot, Marcus Q. Bernardini, Linh T. Nguyen, Pamela S. Ohashi

**Affiliations:** 1 Department of Immunology, University of Toronto, Toronto, Ontario Canada; 2 Princess Margaret Cancer Centre, University Health Network, Toronto, Ontario Canada; 3 Division of Gynecologic Oncology, University Health Network, Toronto, Ontario Canada; 4 Department of Laboratory Medicine and Pathobiology, University Health Network, University of Toronto, Toronto, Ontario Canada; 5 Toronto General Hospital Research Institute, University Health Network, University of Toronto, Toronto, Ontario Canada; 6 Department of Medical Biophysics, University of Toronto, Toronto, Ontario Canada

**Keywords:** B7-H4, B7-H3, B7 family, Epithelial ovarian cancer, Tumor microenvironment, Intrapatient variability, immune heterogeneity

## Abstract

**Background:**

B7-H3 and B7-H4 are highly expressed by many human malignancies making them attractive immunotherapeutic targets. However, their expression patterns and immune contexts in epithelial ovarian cancer have not been well characterized.

**Methods:**

We used flow cytometry, immunohistochemistry, and genomic analyses to determine the patterns of B7-H3, B7-H4, and PD-L1 expression by tumor, stromal, and immune cells in the ovarian tumor microenvironment (TME). We analyzed immune cell frequency and expression of PD-1, TIM3, LAG3, ICOS, TIA-1, granzyme B, 2B4, CD107a, and GITR on T cells; CD20, CD22, IgD, BTLA, and CD27 on B cells; CD16 on monocytes; and B7-H3, B7-H4, PD-L1, PD-L2, ICOSL, CD40, CD86, and CLEC9a on antigen-presenting cells by flow cytometry. We determined intratumoral cellular location of immune cells using immunohistochemistry. We compared differences in immune infiltration in tumors with low or high tumor-to-stroma ratio and in tumors from the same or unrelated patients.

**Results:**

On non-immune cells, B7-H4 expression was restricted to tumor cells whereas B7-H3 was expressed by both tumor and stromal cells. Stromal cells of the ovarian TME expressed high levels of B7-H3 compared to tumor cells. We used this differential expression to assess the tumor-to-stroma ratio of ovarian tumors and found that high tumor-to-stroma ratio was associated with increased expression of CD16 by monocytes, increased frequencies of PD-1^high^ CD8^+^ T cells, increased PD-L1 expression by APCs, and decreased CLEC9a expression by APCs. We found that expression of PD-L1 or CD86 on APCs and the proportion of PD-1^high^ CD4^+^ T cells were strongly correlated on immune cells from tumors within the same patient, whereas expression of CD40 and ICOSL on APCs and the proportion of PD-1^high^ CD8^+^ T cells were not.

**Conclusions:**

This study provides insight into the expression patterns of B7-H3 and B7-H4 in the ovarian TME. Further, we demonstrate an association between the tumor-to-stroma ratio and the phenotype of tumor-infiltrating immune cells. We also find that some but not all immune parameters show consistency between peritoneal metastatic sites. These data have implications for the design of immunotherapies targeting these B7 molecules in epithelial ovarian cancer.

## Background

The B7 family of immunomodulatory proteins provide key costimulatory and coinhibitory signals to T cells. Recently, disruption of interactions between certain B7 family members and their inhibitory binding partners expressed on T cells have shown remarkable clinical success in the treatment of cancer [1, 2]. The first inhibitory interaction to be blocked with clinical success was the binding of inhibitory protein CTLA-4 to prototypical B7 family members CD80 and CD86. CD80 and CD86 expression are upregulated on mature antigen presenting cells (APCs) and provide costimulatory signals to T cells through the binding of CD28. However, CD80 and CD86 preferentially interact with CTLA-4, an inhibitory molecule induced on effector T cells after TCR stimulation and expressed constitutively by T_regs_ [3]. CTLA-4 blocking antibodies have also been shown to facilitate the depletion of T_regs_ [4] in an FcγR-dependent manner in addition to relieving inhibition on the CD28 costimulatory pathway [5]. CTLA-4 blockade provided crucial proof-of-principle validation that checkpoint blockade could augment the anti-tumor response in melanoma.

PD-L1, another member of the B7 family, can inhibit T cells through the binding of PD-1. In addition to being expressed on APCs, PD-L1 can be upregulated on non-hematopoietic tissues such as tumor cells [3]. Importantly, treatment with PD-1/PD-L1 blocking antibodies are better tolerated than treatment with anti-CTLA-4 antibodies [2]. Further, PD-1/PD-L1 blockade has shown activity in a broader range of malignancies [2].

Due to the nondescript symptoms of early stage disease, ovarian cancer is often diagnosed at later stages leading to reduced therapeutic efficacy and success. The development of resistance to platinum-based chemotherapies emerges in 80–90% of ovarian cancer patients, resulting in high rates of relapse and mortality [6]. As a result, the development of treatments that can reduce recurrence will greatly benefit patients with ovarian cancer. Strong evidence that higher T cell infiltration is associated with improved survival [7, 8] indicates that an immune response is mounted against ovarian cancer. For these reasons, use of PD-1/PD-L1 blockade in ovarian cancer is actively being investigated [9–12]. While trials have reported clinical activity, overall response rates have been lower in ovarian cancer than in other malignancies [2]. This could indicate that additional suppressive mechanisms are active in the ovarian TME.

Currently, one priority is to explore new targets that can be used in combination with existing therapies to overcome immunosuppression in the TME. Two potential targets are B7-H3 (CD276; 4Ig-B7-H3; B7RP-2) and B7-H4 (B7x; B7-S1; VTCN1; DD-0110). While the ligand for B7-H3 remains unknown, B7-H4 has been proposed to bind with the Semaphorin 3a/Plexin A4/Neuropilin-1 complex resulting in inhibition of inflammatory CD4^+^ T cell responses and enhanced T_reg_ function [13]. However, Ohaegbulam *et al*. (2017) did not observe interactions between B7-H4 and either Semaphorin 3a or Neuropilin-1 [14] necessitating further investigation into the interaction. B7-H3 and B7-H4 have both been reported to be expressed on immune cells, especially APCs [15, 16]. Unlike prototypical B7 family members CD80 and CD86, B7-H3 and B7-H4 have also been observed to be expressed by a variety of malignancies, making these molecules attractive candidate targets for immunotherapy [15, 16].

Studies in many malignancies including endometrial, cervical, and ovarian cancers have reported an association between higher B7-H3 expression by tumors and poor outcomes [17]. Molecular evidence suggests an association between B7-H3 and the stromal compartment. B7-H3 has been correlated with higher CXCR4, a chemokine receptor important in recruiting fibroblasts to the TME [18, 19], and with a higher stroma score [20] suggesting that B7-H3 may be highly expressed by fibroblasts. Additionally, some studies have evaluated the impact of B7-H3 expression by certain non-immune cell types of the TME. Higher expression of B7-H3 on endothelial cells and tumor vasculature was associated with higher grade malignancies and poor survival [21–25]. B7-H3 expression on a subset of cancer-associated fibroblasts (CAFs) in breast cancer has been reported to contribute to T cell skewing towards regulatory functions [26]. However, studies have not evaluated levels of B7-H3 expression by different cell populations comparatively in the TME.

Clinical trials investigating biologics targeting B7-H3 and B7-H4 are moving forward. Clinical trials testing the safety and efficacy of B7-H3- (NCT02982941, NCT02381314, NCT02923180, NCT02475213) and B7-H4-specific (NCT03514121) antibodies alone and in combination with anti-CTLA-4 or anti-PD-1 are underway. In addition to antibodies, CAR T cells, DARTs (Dual-Affinity Re-Targeting proteins), and antibody-drug conjugates specific for B7-H3 are being explored in clinical trials. A clearer understanding of the expression patterns of B7-H3 and B7-H4 and their associated TMEs will help in informing therapeutic development, choosing the proper therapeutic modality, and designing effective combination therapies.

We have focused on understanding B7-H3 and B7-H4 in the ovarian TME because this malignancy shows the potential to respond to PD-1/PD-L1 blockade, but thus far has shown minimal success. To this end, we used flow cytometry, immunohistochemistry, and genomic analyses to evaluate the TME of epithelial ovarian cancer (EOC). We demonstrate that B7-H4 is primarily expressed by tumor cells, whereas B7-H3 is expressed by both tumor and stromal cells. Furthermore, we report that stromal cells express B7-H3 at higher levels than tumor cells in the ovarian TME and find that the tumor-to-stroma ratio (T:S) impacts the average level of B7-H3 expression in a tumor and is associated with differences in the phenotype of infiltrating immune cells.

## Methods

### Tumor and blood specimens

All human tissues and bloods were obtained through protocols approved by the institutional review board (University Health Network Research Ethics Board). Surgical specimens were obtained from the UHN Biospecimen Program. Written informed consent was obtained from all donors.

### Tumor digestion

Tumors were mechanically dissociated into pieces less than 1 mm in diameter and resuspended in enzymatic digestion media consisting of IMDM (Lonza) supplemented with 1 mg/mL collagenase type IV (Sigma), 10 μg/mL DNase I (Pulmozyme, Roche), 100 units/mL penicillin, 100 μg/mL streptomycin (Lonza), 10 μg/mL gentamicin sulfate (Lonza), 2 mM L-glutamine (Lonza), and 1.25 μg/mL amphotericin B. Tumor suspension was incubated for two 30 min incubations under rotation at 37 °C with mechanical dissociation on the gentleMACS dissociator (Miltenyi) using the programs for soft human tumors before, in between, and after the incubations. Single cell suspension was washed 3 times with wash media consisting of PBS supplemented with 10% FCS, 100 units/mL penicillin, and 100 μg/mL streptomycin (Lonza). All centrifugations were done at slow speed (330 x g).

### Ex vivo flow cytometry staining on tumor samples after enzymatic digestion

Staining was completed at 4 °C. Fc receptors were blocked with Fc block (BD) or media supplemented with 10% human serum for 30 min prior to staining for surface expression. Stained cells were washed with PBS and stained with fixable viability dye (Thermo Fisher Scientific) according to manufacturer’s protocol. Cells were washed with FACS buffer and fixed in 2% paraformaldehyde for 30 min. Intracellular staining for TIA-1 and GzmB was performed after fixation with 2% paraformaldehyde using permeabilization buffer (Thermo Fisher Scientific) according to the manufacturer’s protocol. Staining of the immune infiltrate was done on fresh tumor samples directly after digestion. Staining for expression of epithelial and stromal markers on tumor and stromal cells was performed on viably-frozen tumor samples. Calculation of T:S ratio by differential B7-H3 expression was done of fresh samples as the freeze-thaw process impacted survival of these cell types and therefore resulted in alterations to the T:S. Antibodies used are listed in Additional file 1: **Table S1**.

### Flow cytometric gating strategies

For staining of fresh samples from ovarian cancer samples, markers that exhibited a negative population (lineage-defining markers, total expression of T cell inhibitory and activation markers) were gated according to patient-matched fluorescence minus one controls (FMO). For markers that exhibited shifts in expression levels (CD16 expression on monocytes, APC inhibitory and activating markers), gMFI was normalized to control PBMCs from a hemochromatosis donor draw run in parallel according to the following formula: (gMFI_sample_ – gMFI_FMO_)/(gMFI_PBMCs_). These measures were taken to control for variability introduced due to tumor sample processing, staining, and data acquisition being completed on different days as a result of the requirement for fresh tissue.

### Immunohistochemical staining

Tumor specimens were fixed in 10% formalin solution (VWR), processed, and embedded in paraffin. Sections (4.5 μm) were dewaxed, rehydrated, and peroxidase activity was blocked with 3% hydrogen peroxide solution. In cases where the antibody clone used to detect an antigen was changed, 3 sample cases were stained with both antibody clones to ensure consistency in the results. Antigen was retrieved with heat treatment and either 10 mM sodium citrate (pH 6.0) [anti-B7-H3 (clone SP206), anti-CD8 (clone C8/144B), anti-CD3 (clone 2GV6), anti-CD20 (clone EP459Y or L26), anti-FoxP3 (clone mAb22510 or 236A/E7)], Tris-EDTA (pH 9.0) [anti-CD8 (clone4B11), anti-B7-H4 (D1M8I)], or 1% pepsin (pH 2.0) [anti-CD3 (polyclonal)] prior to incubation in blocking solution. Slides were scanned using a Nanozoomer 2.0HT (Hamamatsu Photonics) and cell quantifications (CD3, CD8, FoxP3, CD20, CD68) and T:S (H&E) were calculated using Halo analysis software (v2.0.1145.14).

### Scoring of immune cell infiltration density

Stained slides were visually assessed and scored on a 5-point scale for the level of immune cell infiltration into epithelial or stromal areas in relation to the range of infiltration in the stained cohort according to the following scale:

1 – no positive events found on slide.

2 – rare positive events observed.

3 – low density of infiltration.

4 – medium density of infiltration.

5 – high density of infiltration.

### Statistical analysis

Univariate linear regressions and likelihood-ratio tests were used to interrogate the TCGA ovarian serous cystadenocarcinoma dataset, and aggregate analysis of gene expression across 22 TCGA datasets were performed using cBioPortal [27, 28] and R software Version 3.4.0. Spearman rank correlations were computed between B7-H3 and all other genes. Correlations in each dataset were Z-score-normalized, and Stouffer’s method was used to generate a combined Z-score for each gene across all 22 datasets. Genes were then ranked by the combined Stouffer Z-score.

Paired T tests, Mann-Whitney U tests, and Mantel-Cox tests were performed using GraphPad Prism Version 5.0c. Two-sided *p* values of < 0.05 were deemed to be significant (*p < 0.05; **p < 0.01; ***p < 0.001; ns *p* > 0.05).

## Results

### B7-H3 and B7-H4 have different expression patterns in EOC

**Fig. 1:**
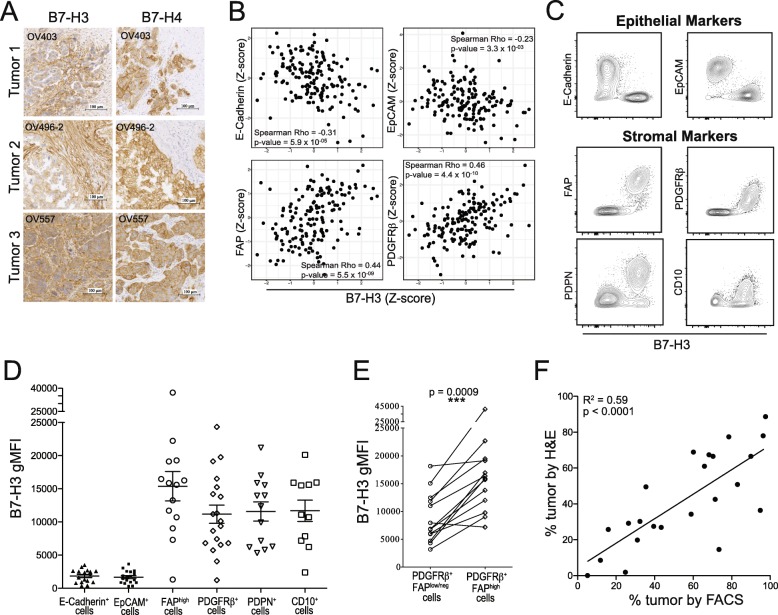
B7-H3 expression level differentiates between tumor and stromal compartments in EOC. A: B7-H3 (left) and B7-H4 (right) immunohistochemical staining. B: Linear correlations between Z-scores of protein levels of B7-H3 and epithelial cell markers (E-Cadherin, EpCAM) or stromal cell markers (FAP, PDGFRβ) in the TCGA ovarian serous cystadenocarcinoma dataset. C: Flow cytometry staining of CD45^−^ cells from EOC tumors for B7-H3, epithelial markers (E-Cadherin, EpCAM), and stromal markers (FAP, PDGFRβ, PDPN, CD10). D: B7-H3 surface expression (gMFI) on cell populations positive for epithelial cell markers (E-Cadherin, EpCAM) or stromal cell markers (FAP, PDGFRβ, PDPN, CD10). E: Comparisons between B7-H3 expression on PDGFRβ^+^FAP^low/neg^ and PDGFRβ^+^FAP^high^ cells. Paired values are from the same patient. Significance was determined by paired T test (*p* = 0.0009). F: Linear correlation between T:S defined by histology (H&E staining) and flow cytometry (B7-H3 staining) (R^2^ = 0.59; *p* < 0.0001). Data from C, D, E are pooled from 2 experiments

To evaluate expression of B7-H3 and B7-H4 by different cell types in the ovarian TME, we stained epithelial ovarian tumors (Additional file 1: Table S2) using immunohistochemistry (IHC) and flow cytometry. By IHC, B7-H3 was expressed by both tumor and stromal cells in the epithelial ovarian TME (*n* = 39) (Fig. 1A). In contrast, B7-H4 expression was restricted to the tumor cell compartment (Fig. 1A). While all EOC cases examined had some level of cytoplasmic B7-H4 expression (*n* = 28), a minority of tumors analyzed (10/25) exhibited high levels of surface B7-H4 expression by flow cytometry. In contrast, most non-immune cells in EOC exhibited membranous staining for B7-H3 (28/28). This demonstrated that B7-H3 and B7-H4 have different expression patterns by cells of the ovarian TME.

To assess whether B7-H3 protein expression was restricted to the TME, we stained 4 non-cancerous tissues for B7-H3. Liver, tonsil, and spleen all exhibited cytoplasmic but not membranous B7-H3 staining. However, extravillous trophoblasts of the placenta stained strongly for membranous B7-H3 expression (Additional file 2: Fig. S1). These results are in accordance with previous reports [22, 29–31] and suggest that B7-H3 expression, while restricted in non-cancerous tissues, may be readily induced in the appropriate environment.

To further investigate the level of B7-H3 and B7-H4 expression on tumor and stromal cells we stained CD45^−^ cells by flow cytometry. We identified two separate populations that expressed different levels of B7-H3. Because B7-H4 expression was restricted to the tumor cell compartment (Fig. 1A), we identified the B7-H3^low^B7-H4^+^ population as tumor cells (red) and the B7-H3^high^B7-H4^neg^ population as stromal cells (blue) (Additional file 2: Fig. S2A). Both tumor and stromal cells were positive for surface B7-H3 expression (*n* = 28); however, tumor cells consistently expressed lower levels of B7-H3 compared to stromal cells (Additional file 2: Fig. S2B). In contrast, B7-H4 was expressed by tumor cells but not by stromal cells (Additional file 2: Fig. S2C). This demonstrates that B7-H3 and B7-H4 exhibit different patterns of expression by tumor and stromal cells of the EOC TME.

To determine differences in HLA class I expression by tumor and stromal cells in EOC, we examined these populations for HLA-ABC expression by flow cytometry. Tumor and stromal cells expressed different levels of HLA-ABC, with stromal cells (blue) expressing higher levels than tumor cells (red). Notably, surface expression of B7-H3 and HLA-ABC appeared to be positively correlated on stromal cells (Additional file 2: Fig. S2D).

### Level of B7-H3 expression can be used to distinguish tumor from stromal cells

To assess which genes are most strongly associated with B7-H3 expression, we performed rank analysis of the 50 genes with the most significant positive correlation with B7-H3 expression across 22 TCGA datasets. Strikingly, half of the top 50 genes (denoted in red) correlated with higher B7-H3 expression had roles associated with stromal cells and extracellular matrix (ECM) remodeling such as collagens, matrix metalloproteinases, and lysyl oxidase cross-linking enzymes (Additional file 2: Fig. S3). These data suggest that high B7-H3 expression results from greater levels of stromal content across multiple tumor types.

To further investigate the link between B7-H3 expression and stromal cell content in ovarian cancer, we interrogated the TCGA dataset of ovarian serous cystadenocarcinoma samples. We found that B7-H3 protein expression was strongly associated with expression of stromal markers fibroblast activation protein alpha (FAP) and PDGFRβ, and negatively associated with epithelial markers EpCAM and E-Cadherin (Fig. 1B), demonstrating that B7-H3 shows strong positive correlations with stromal markers and negative associations with epithelial markers.

To confirm that stromal cells express higher levels of B7-H3, we examined B7-H3 staining intensity by flow cytometry (gMFI) of CD45^−^ cells expressing epithelial (tumor) or stromal markers (Additional file 2: Fig. S4). Cells positive for epithelial markers E-Cadherin or EpCAM expressed low levels of B7-H3 while cells positive for stromal markers FAP, PDGFRβ, podoplanin (PDPN), or CD10 expressed strikingly high levels of B7-H3 (Fig. 1C,D; Additional file 2: Fig. S5A,B). FAP^high^ and PDGFRβ^+^ cells had average B7-H3 gMFIs above that of EpCAM^+^ cells of 14,147 and 9610, respectively. These data demonstrate that stromal cells in the ovarian TME express higher surface levels of B7-H3 than tumor cells.

To investigate if CAFs differentially express B7-H3, we compared B7-H3 gMFI on cells expressing high levels of the CAF marker FAP to B7-H3 gMFI on cells positive for PDGFRβ but expressing low levels of FAP. While PDGFRβ is also upregulated by CAFs, it is expressed at lower levels by other stromal cells [32]. Consistent with reported expression patterns, we found that PDGFRβ was expressed by a larger proportion of stromal cells than FAP (Additional file 2: Fig. S5C). We compared proportions of PDGFRβ^+^FAP^high^ to the total proportion of FAP^high^ cells and found that the proportions were not significantly different (Additional file 2: Fig. S5D) indicating that all FAP^high^ cells coexpressed PDGFRβ. PDGFRβ^+^FAP^high^ cells expressed B7-H3 at significantly higher levels than compared to PDGFRβ^+^FAP^low/neg^ cells (Fig. 1E). This indicates that CAFs express higher levels of B7-H3 than other tumor-associated stromal cells.

Given the wide differential in levels of B7-H3 expression on tumor and stromal cells (Fig. 1D; Additional file 2: Fig. S5A,B), we explored whether it alone could be used to identify the tumor and stromal components of the CD45^−^ fraction by flow cytometry. We compared the proportion of tumor cells calculated from H&E stained slides (Additional file 2: Fig. S6A) and the proportion of tumor cells (B7-H3^low^) as assessed by flow cytometry (Additional file 2: Fig. S6B) (*n* = 23). We found a strong and highly significant correlation (Fig. 1F; R^2^ = 0.59, *p* < 0.0001) indicating that B7-H3 can be used to assess the T:S by flow cytometry.

Higher stromal content of tumors has been associated with poor overall and disease-free survival [33]. To assess if T:S was significantly associated with differences in disease progression or survival we compared time to recurrence or death for patients with tumors that had low (blue; T:S < 1.5) or high (orange; T:S > 1.5) T:S and found that patients with high T:S trended towards having longer recurrence-free survival (Additional file 2: Fig. S7; *p* = 0.098) suggesting that stromal content may impact recurrence in epithelial ovarian cancer.

### B7-H4 and PD-L1 are differentially expressed by tumor and stromal cells

**Fig. 2:**
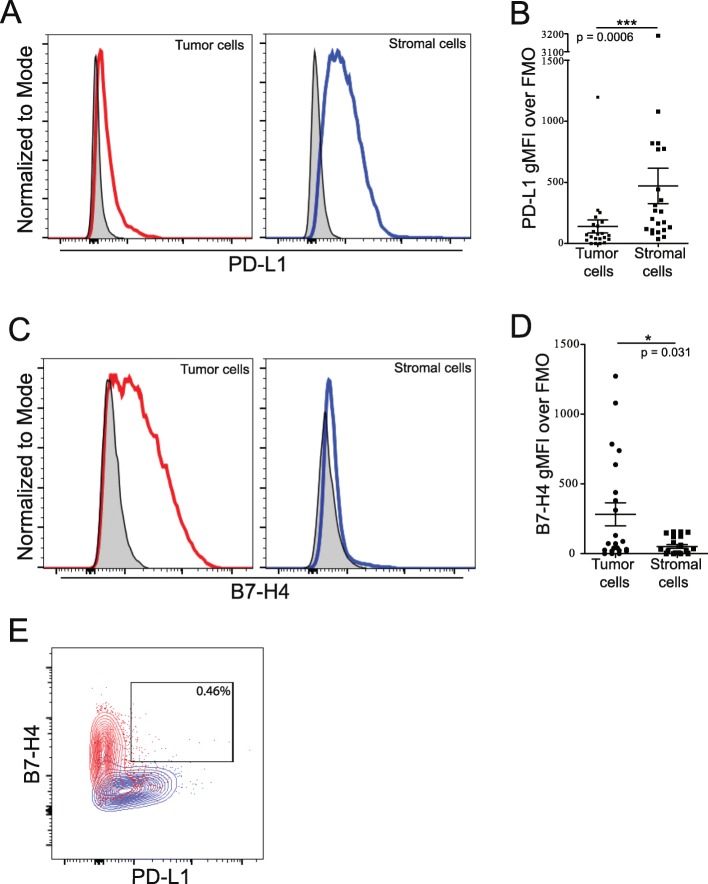
Cells expressing both PD-L1 and B7-H4 are not prominent within the CD45 negative population. Single cell suspensions from freshly isolated tumor cells were stained for CD45, B7-H3, PD-L1 and B7-H4. Tumor and stromal cells were gated from the CD45- population based on level of B7-H3 expression. A: Examples of PD-L1 staining on tumor (left) and stromal (right) cells are shown. B: Level of PD-L1 expression on tumor and stromal populations (Mean ± SEM; *p* = 0.0006). C: Examples of B7-H4 staining on tumor (left) and stromal (right) cells. D: Level of B7-H4 expression on tumor (left) and stromal (right) populations (Mean ± SEM; *p* = 0.031). E: Example plot showing frequency of B7-H4^+^PD-L1^+^ positivity of total CD45^−^ cells. Tumor cells shown in red, stromal cells shown in blue, matched FMOs are shown in grey. Statistical significance was determined by Mann Whitney U test

We used flow cytometry to differentiate tumor and stromal cells by level of B7-H3 expression to gain more insight into the expression of B7 family members PD-L1 and B7-H4 in the ovarian TME. Our analysis showed that PD-L1 and B7-H4 are predominantly expressed by different cell populations in the ovarian TME. PD-L1 was more highly expressed in the stromal rather than tumor compartment (Fig. 2A,B; *p* = 0.0006) while B7-H4 expression was primarily expressed by the tumor compartment (Fig. 2C,D; *p* = 0.031). Importantly, we did not observe a B7-H4^+^PD-L1^+^ double positive population (Fig. 2E). These data show that B7 family members are differentially expressed by tumor and stromal cell populations in the ovarian TME.

### T:S is not associated with frequency of infiltrating immune cells

**Fig. 3:**
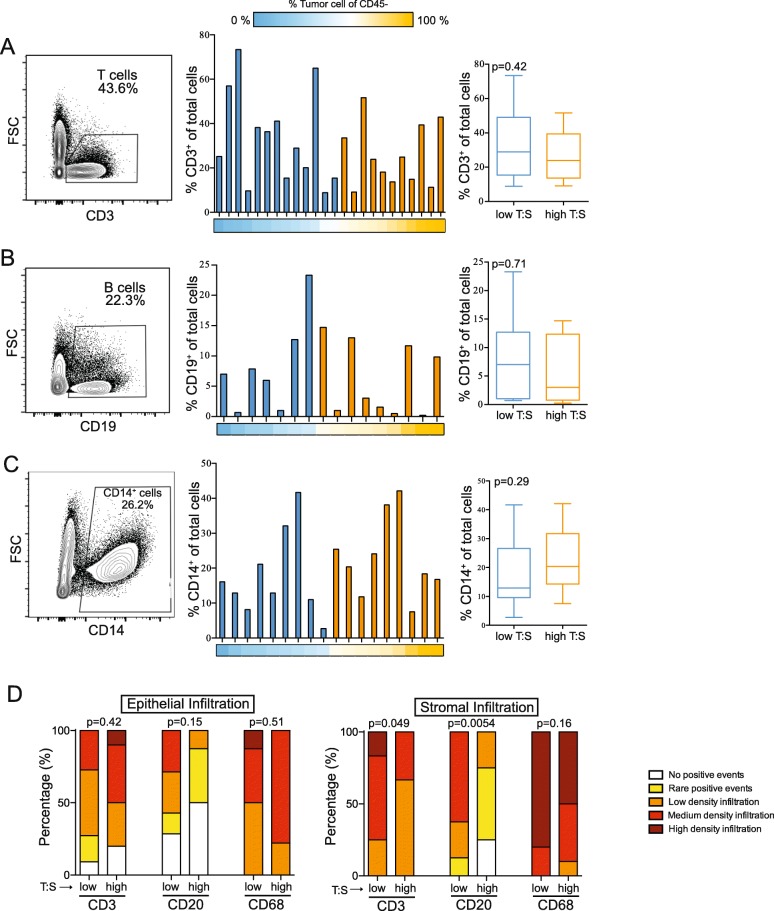
Higher tumor-to-stroma ratio (T:S) does not significantly correlate with higher proportions of infiltrating immune cells. A, B, C: Frequencies of T cells (A; *n* = 24), B cells (B; *n* = 16), and monocytes (C; *n* = 18) infiltrating EOC tumors. D: Infiltration scores of tumor-infiltrating CD3^+^, CD20^+^, and CD68^+^ cells in the epithelial or stromal cell compartments. T:S was determined by flow cytometry using B7-H3 staining and is represented below bar graphs by color gradient. Whisker and box plots summarize data from tumors with low (T:S < 1.5; blue) or high (T:S > 1.5; orange) T:S. Statistical significance was determined by Mann Whitney U test

To determine if the proportion of stromal cells in the ovarian TME impacted the frequency of immune cell infiltration, we compared immune cell frequency in tumors with low (blue; T:S < 1.5) or high (orange; T:S > 1.5) T:S. We did not observe significant differences in the frequency of T cells (*n* = 24; Fig. 3A), B cells (*n* = 16; Fig. 3B), or CD14^+^ cells (*n* = 18; Fig. 3C) between these two groups suggesting that the T:S does not significantly impact the recruitment of immune cells.

To determine if there were differences in the localization of infiltration, histology sections were stained for immune markers and scored for the extent of infiltration in the epithelial or stromal tumor compartments based on the density of positive staining for immune cell populations. T:S was not associated with differences in T cell (CD3^+^), B cell (CD20^+^), or macrophage (CD68^+^) infiltration density in the epithelial tumor compartment; however, T and B cells exhibited significantly higher densities of infiltration in the stromal compartment of tumors with higher stromal content (Fig. 3D). These data suggest that, while detectable differences in immune cell recruitment to tumors is not corelated with T:S, local density of infiltration may be affected by tumor and stromal composition of the ovarian TME.

### CD8^+^ T cells from tumors with high T:S express higher levels of PD-1

**Fig. 4:**
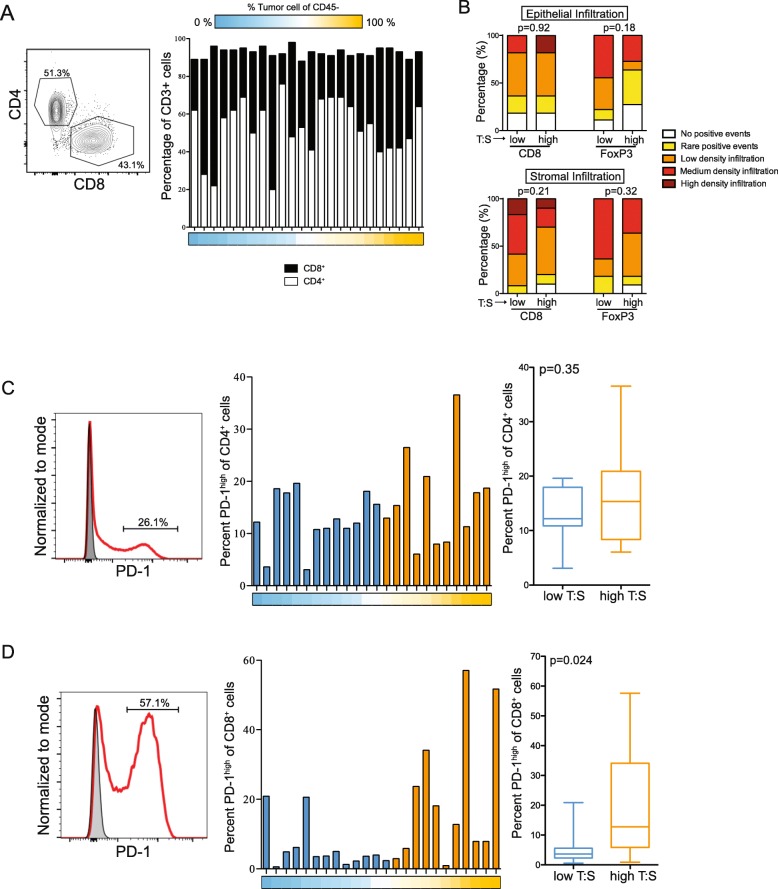
Tumors with a higher tumor-to-stroma ratio (T:S) have an increased proportion of infiltrating CD8^+^ T cells expressing high levels of PD-1. A: Proportions of CD4^+^ and CD8^+^ T cells isolated from tumors (n = 24). B: Infiltration scores of tumor-infiltrating CD8^+^ and FoxP3^+^ cells in tumors with low (T:S < 1.5; blue) or high (T:S > 1.5; orange) T:S. C,D: Proportion of CD4^+^ (C) and CD8^+^ (D) T cells (n = 24) expressing high levels of PD-1. Whisker and box plots summarize data from tumors with low (T:S < 1.5; blue) or high (T:S > 1.5; orange) T:S. T:S of tumor is represented below by color gradient (A,C,D). Statistical significance was determined by Mann Whitney U test

Further analysis was done to evaluate whether there were differences in T cell phenotype associated with T:S. We did not find differences in proportions of infiltrating CD4^+^ or CD8^+^ T cells in relation to the T:S (*n* = 24; Fig. 4A). We also did not see differences in the density of infiltration of cytotoxic (CD8^+^) or regulatory (FoxP3^+^) T cells in either the tumor or stromal cell compartments (Fig. 4B).

To further interrogate the phenotype of the infiltrating T cells, we analyzed expression of PD-1 on tumor-infiltrating CD4^+^ and CD8^+^ T cells. While some level of PD-1 expression on T cells was observed in all tumors examined, many samples exhibited a distinct PD-1^high^ population with a bimodal distribution in both CD4^+^ and CD8^+^ T cell populations (n = 24; Fig. 4C,D). No significant differences were seen in the frequency of PD-1^high^ CD4^+^ cells in relation to T:S (Fig. 4C), but tumors with a higher T:S had significantly higher proportions of infiltrating CD8^+^ T cells exhibiting high levels of PD-1 (Fig. 4D). However, we did note that 2/13 samples with low T:S had high frequencies of CD8^+^PD-1^high^ cells, and 5/11 samples with high T:S had low frequencies of CD8^+^PD-1^high^ cells. This could indicate that T cell functionality or exhaustion may be influenced by T:S but additional factors are likely contributing to this T cell phenotype.

To investigate if neoadjuvant chemotherapy (NACT) contributed to differences in PD-1 expression by T cells, we compared frequencies of PD-1^high^ CD4^+^ and CD8^+^ T cells between patients who were and were not treated with NACT. We did not find any associations between proportion of T cells expressing high levels of PD-1 and chemotherapy treatment indicating that the observed phenotype is independent of chemotherapy treatment (Additional file 2: Fig. S8A).

To investigate other possible differences in the phenotype of T and B cells infiltrating tumors with high or low T:S, we analyzed expression of various markers of activation. We did not observe significant differences in the proportion of CD4^+^ or CD8^+^ T cells co-expressing high levels of PD-1 and exhaustion markers TIM3 or LAG3 (Additional file 2: Fig. S9A,B). Similarly, we did not observe significant differences in expression of activation or cytotoxicity markers examined (ICOS, TIA-1, granzyme B, 2B4, CD107a, GITR) (Additional file 2: Fig. S9C,D).

To further interrogate the phenotype of infiltrating B cells, we stained for several B cell markers associated with naïve (CD20^+^CD22^+^; IgD^+^) or antigen-experienced (BTLA^+^; CD27^+^) status (Additional file 2: Fig. S10A). We did not observe differences in proportion of CD19^+^ B cells expressing BTLA, CD27, CD20 and CD22, or IgD in relation to T:S (Additional file 2: Fig. S10B).

We found that tumors with higher T:S had greater frequencies of CD8^+^ T cells expressing high levels of PD-1; however, we did not find differences in T cells co-expressing inhibitory markers TIM3 or LAG3 with high levels of PD-1. Additionally, we didn’t find differences in proportions of T cells expressing ICOS, TIA-1, granzyme B, 2B4, CD107a, or GITR, nor in proportions of B cells expressing CD20, CD22, BTLA, or CD27. Taken together, this in-depth analysis of surface marker expression revealed that T and B cells isolated from the ovarian TME express variable levels of inhibitory and activating markers, and the frequency of PD-1^high^ CD8^+^ T cells was greater in tumors with higher T:S.

### Tumors with higher T:S are associated with changes in the phenotype of monocytes and APCs

**Fig. 5:**
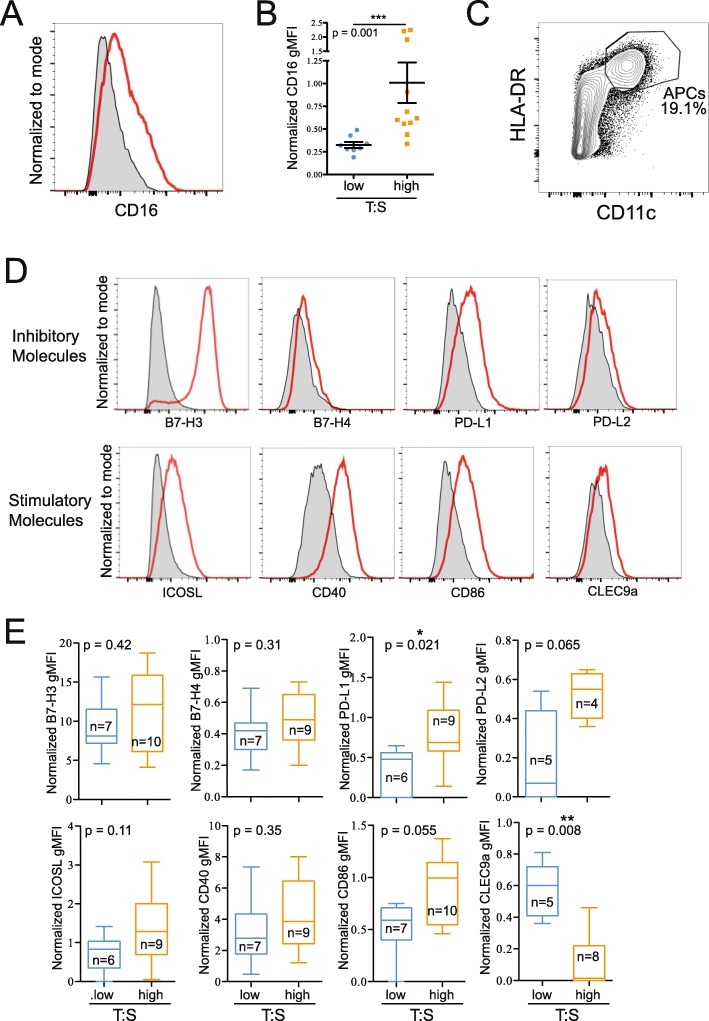
Tumors with a higher tumor-to-stroma ratio (T:S) have increased CD16 expression on infiltrating monocytes (CD14^+^), and mature APCs (CD11c^+^HLA-DR^high^) expressing higher levels of PD-L1 and lower levels of CLEC9a. A: Histogram showing representative CD16 staining on monocytes (red) compared to FMO (grey). B: Differences in normalized CD16 expression compared to FMO on monocytes from tumors with low (T:S < 1.5; blue) or high (T:S > 1.5; orange) T:S. C: Flow plot showing representative gating of mature APCs. D, E: Mature APCs were stained for activating (ICOSL, B7-H3, CD40, CD86, CLEC9a) and inhibitory (B7-H4, B7-H3, PD-L1, PD-L2) surface molecules. Histograms show representative staining (red) compared to FMO (grey) (D). E: Normalized surface molecule expression on mature APCs from tumors with low (T:S < 1.5; blue) or high (T:S > 1.5; orange) T:S. Statistical significance was determined by Mann Whitney U test

To further characterize the immune infiltrate of ovarian cancer, we examined the phenotypes of infiltrating monocytes (CD14^+^) and mature APCs (CD11c^+^HLA-DR^high^). Monocytes from tumors with a high T:S expressed higher levels of CD16 (Fig. 5A,B). Mature APCs isolated from tumors (Fig. 5C) were stained for coinhibitory (B7-H3, B7-H4, PD-L1, PD-L2) and costimulatory (B7-H3, ICOSL, CD40, CD86, CLEC9a) molecules (Fig. 5D). B7-H3 was highly expressed on APCs from all patients, but no differences in level of expression were seen in relation to the T:S. Similarly, no significant differences were seen in B7-H4, ICOSL, CD40, PD-L2 or CD86 expression in relation to T:S, although expression of both PD-L2 and CD86 trended towards being more highly expressed on APCs infiltrating tumors with higher T:S. APCs isolated from tumors with higher T:S expressed significantly higher levels of inhibitory PD-L1 and significantly lower expression of CLEC9a (Fig. 5E). We confirmed that these differences in monocyte and APC phenotype were not associated with NACT treatment (Additional file 2: Fig. S8B,C). These data indicate that when a higher proportion of tumor cells are present, monocytes express higher levels of CD16 and APCs express higher levels of PD-L1.

### Heterogeneity in immune cells isolated from different metastatic sites

**Fig. 6:**
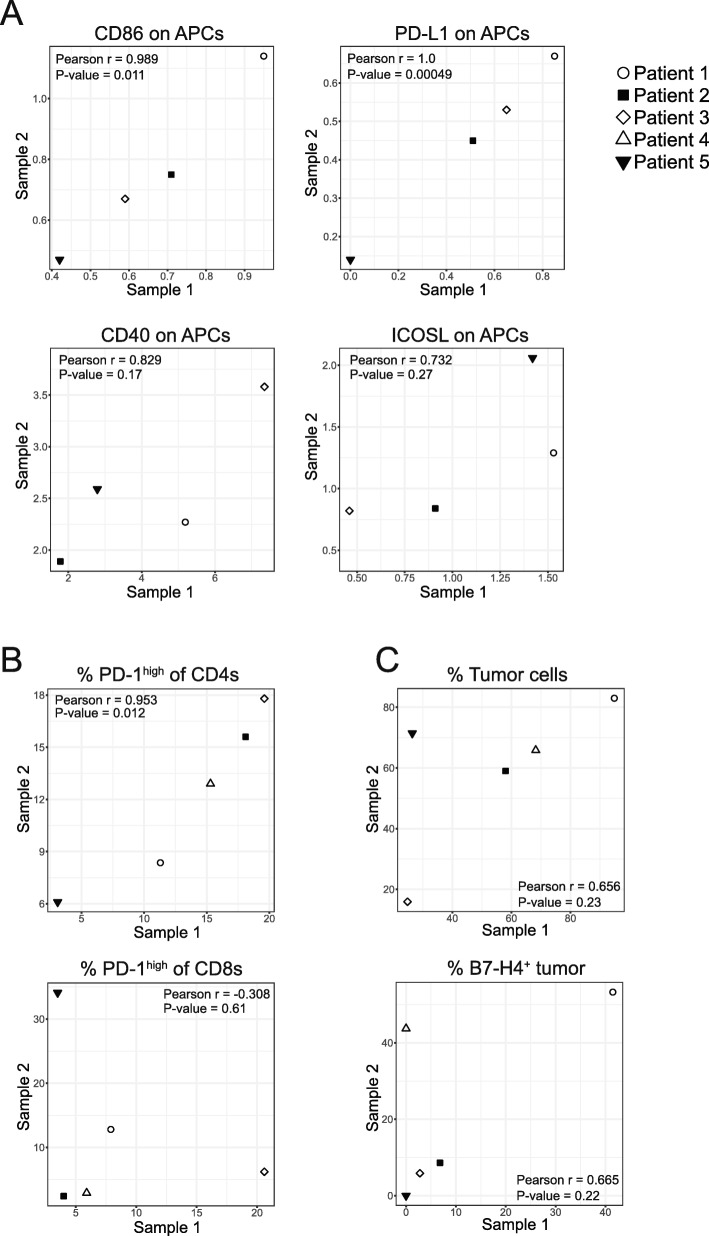
Tumors from the same patient show strong correlation across expression of some immune features. A: Linear correlations between normalized expression of CD86 (r = 0.989; *p* = 0.011), PD-L1 (r = 1.0; *p* = 0.00049), CD40 (r = 0.829; *p* = 0.17), and ICOSL (r = 0.732; *p* = 0.27) on APCs (CD11c^+^HLA-DR^high^) isolated from two tumors within the same patient. B: Linear correlations between proportion of CD4^+^ (r = 0.953; *p* = 0.012) or CD8^+^ (r = − 0.308; *p* = 0.61) T cells isolated from two tumors within the same patient expressing high levels of PD-1. C: Linear correlations between the percentage of non-immune cells that are tumor cells (r = 0.656; *p* = 0.23) and the proportion of tumor cells expressing B7-H4 (r = 0.665; *p* = 0.22)

For 5 patients, we received two tumor samples from different metastatic sites (ovary and omentum [*n* = 4], or right and left ovary [*n* = 1]). We compared data from intrapatient tumors to see if immune cell frequency or phenotype was consistent within a patient or governed by tumor-specific parameters. We calculated the linear correlation between levels of expression of select markers isolated from different tumors from the same patient and found that expression of CD86 on APCs (r = 0.989; *p* = 0.011), PD-L1 on APCs (r = 1; *p* = 0.00049) (Fig. 6A), and the proportion of PD-1^high^ CD4^+^ T cells (r = 0.953; *p* = 0.012) (Fig. 6B) all exhibited strong positive correlations between tumor sites. Other parameters such as expression of CD40 on APCs (r = 0.829; *p* = 0.17), ICOSL on APCs (r = 0.732; *p* = 0.27) (Fig. 6A), the proportion of PD-1^high^ CD8^+^ T cells (r = − 0.308; *p* = 0.61) (Fig. 6B), the proportion of tumor cells (r = 0.656; *p* = 0.23), and the proportion of B7-H4-expressing tumor cells (r = 0.665; *p* = 0.22) (Fig. 6C) were not significantly correlated between tumor sites. These data demonstrate that the level of expression of PD-L1 and CD86 on APCs and the proportion of PD-1^high^ CD4^+^ T cells are strongly correlated between metastatic sites within a patient but expression of CD40 and ICOSL on APCs, the proportion of PD-1^high^ CD8^+^ T cells, the proportion of tumor cells, and the proportion of B7-H4-expressing tumor cells were not correlated between metastatic sites.

To examine whether the site of metastatic disease influenced immune cell frequency we compared T, B, and monocyte frequencies from ovary and omental tumors. We found that omental tumors had higher frequencies of T and B cells (Additional file 2: Fig. S11A,B). Monocyte frequency trended towards being lower in omental tumors (Additional file 2: Fig. S11C) which could be due to the increase in the T and B cell populations. Despite finding a higher frequency of T cells in omental tumors, we did not observe differences in the proportion of T cells that were CD4^+^ or CD8^+^ (Additional file 2: Fig. S11D).

To interrogate whether the site of metastatic disease influenced immune cell phenotype we compared cells isolated from ovary or omental tumors. We did not find differences in frequency of PD-1^high^ T cells (Additional file 2: Fig. S11E), level of CD16 expression on monocytes (Additional file 2: Fig. S11F), or expression of activating and inhibitory markers on APCs (Additional file 2: Fig. S11G) between the two metastatic sites. These data indicate that while immune cell frequency is influenced by the metastatic site, the organ site does not influence immune cell phenotype.

## Discussion

### Cell type-specific expression of B7-H3, B7-H4, and PD-L1 in the ovarian TME

B7-H3, B7-H4, and PD-L1 have all been reported to be upregulated in human malignancies but their co-expression patterns in EOC has not previously been reported. We found that B7-H4 was expressed by tumor cells and at low levels on tumor-infiltrating APCs (Fig. 1A; Fig. 2C,D; Fig. 5D,E), but was not observed to be expressed by stromal cells (Fig. 1A; Fig. 2C,D; Additional file 2: Fig. S2C). The expression of B7-H4 on the cell surface of tumor cells provides opportunities for therapeutic targeting using T cells expressing chimeric antigen receptors. This strategy is currently being investigated in pre-clinical models [34]. Combination of B7-H4-targeting therapies with therapies that target the tumor stroma could improve efficacy of anti-B7-H4 drugs given the role of the tumor stroma in potentiating immunosuppression [35] and restricting immune infiltration [36].

PD-L1 also had a restricted expression pattern; however, in contrast to B7-H4 this marker was primarily expressed on stromal cells in EOC (Fig. 2A,B). As a result, B7-H4 and PD-L1 were not co-expressed (Fig. 2E). PD-L1 was expressed on tumor-infiltrating APCs at higher levels than B7-H4. This differential expression pattern suggests that combination therapies against both molecules will target different cell populations in the ovarian TME offering opportunities for additive or synergistic effects.

In contrast to both B7-H4 and PD-L1, B7-H3 was broadly expressed by many cell types in EOC including tumor cells, stromal cells, and APCs (Figs. 1, 5). As a result, therapies specific for B7-H3 could target multiple cell populations. Furthermore, the high levels of B7-H3 expression on tumor stroma (Fig. 1) and tumor-infiltrating APCs (Fig. 5) in addition to some expression by non-cancerous tissues (Additional file 2: Fig. S1) suggest that B7-H3 expression on non-tumor cells can be induced given the appropriate environment. Inducible expression of B7-H3 might lead to an increase in on-target off-tumor toxicities and should be considered when designing therapies against B7-H3.

A report by Costa *et al*. [26] identified four fibroblasts subsets in the TME of human breast cancer. High frequencies of one CAF subset characterized by high levels of FAP expression was associated with an accumulation of FoxP3^+^ T lymphocytes in tumors. Isolation of this CAF subset and subsequent coculture with T cells lead to increased polarization to T_regs_, which could be partially inhibited through knockdown of B7-H3 expression [26]. We also found that fibroblasts in the ovarian TME expressed B7-H3, but their association with T_regs_ remains to be explored.

In this study, we have used flow cytometry to quantify the level of marker expression on the cell surface and compare the level of expression on different cell types. Further, enzymatic digestion allows for analysis of a larger tumor, which decreases the effect of sampling bias. However, this method loses spatial information and tumor architecture. Other groups have examined coexpression of B7 molecules using other methods such as quantitative immunofluorescence on tissue microarrays [37]. These methods have the advantage of retaining information about tumor architecture and cellular proximity; however, fewer markers were analyzed and sampling bias may be stronger given the smaller amount of tissue analyzed. As a result of the different limitations, these methods will be complementary in constructing an accurate picture of the TME.

### Reinterpreting clinical correlations in light of higher B7-H3 expression by stromal cells

High expression of B7-H3 on stromal cells could contribute significantly to its association with negative clinical prognostic factors in cancers. Evaluation of whole tumors by RNA-Seq yields average expression levels and cannot distinguish cell type-specific differential expression. The high level of B7-H3 expression on stromal cells (Fig. 1A,B,C,D) will impact the total expression of B7-H3 in a tumor and supports the interpretation that elevated B7-H3 in the TME correlates with an increased prevalence of stromal cells in the tumor. Given the association of fibroblast signatures with poor outcomes [38, 39] and chemoresistance [40], the association of B7-H3 expression with poor prognosis could be related to its association with higher stromal content in situations where cell types are not taken into account. Furthermore, particularly high levels of B7-H3 expression on CAFs (Fig. 2E) could result in the presence of higher proportions of protumorigenic CAFs increasing the total B7-H3 levels. As a result, high total tumoral levels of B7-H3 expression could reflect higher stromal content and higher CAF frequency in addition to higher levels of immune inhibition, all of which could contribute to its association with poor prognosis.

### Higher T:S is associated with higher CD16 expression on infiltrating monocytes, higher PD-L1, and lower CLEC9a expression on infiltrating APCs

Monocytes from tumors with a higher T:S expressed higher levels of CD16 (Fig. 5A,B). CD16-expressing monocytes are found at higher proportions in the peripheral blood of patients with gastrointestinal carcinoma [41] and patients with metastatic melanoma who respond to ipilimumab treatment [42]. CD16 expression has been shown to be upregulated on monocytes by TNFα [43, 44] and suppressed by miR-218 [45] possibly through its repression of TNFR1-mediated NF-κB activation [46]. Therefore, higher CD16 expression is consistent with a more proinflammatory cytokine milieu. CD14^+^CD16^+^ intermediate monocytes have been shown to express high levels of cytokines such as TNFα [47, 48]. As a result, these cells could contribute to the proinflammatory cytokine milieu and could augment CD16 expression in an autocrine manner. However, CD16 can also be liberated from the cell surface by ADAM17 [49], a sheddase responsible for TNFα activation [50]. Therefore, it is unclear if upregulation of CD16 is a direct or indirect effect of TNFα expression in this context.

CD16-expressing monocytes may also contribute to anti-cancer immunity through antibody-dependent cellular cytotoxicity. In *in vitro* cultures, CD16-expressing monocytes isolated from PBMCs of patients with melanoma were able to lyse T_regs_ when given ipilimumab [42]. Additionally, responders to ipilimumab had a reduction in the percentage of FoxP3^+^ cells within the tumor post-treatment with ipilimumab [42] indicating that CD16-expressing monocytes may contribute to intratumoral T_reg_ depletion in vivo. A higher frequency of CD16^+^ monocytes could suggest a predisposition to respond to antibody therapies that utilize Fc regions with higher affinity for CD16.

Tumors with higher T:S have increased frequency of infiltrating CD8^+^ T cells expressing high levels of PD-1 (Fig. 4D), increased PD-L1 expression and decreased CLEC9a expression on infiltrating mature APCs (Fig. 5D,E). Additionally, expression of CD86 and PD-L2 trended towards being higher in tumors with higher T:S. In humans, CLEC9a is expressed by a subpopulation of DCs which are capable of cross-presenting to CD8^+^ T cells [51]. CLEC9a is a C-type lectin receptor that facilitates antigen uptake by these DCs but is quickly downregulated after antigen uptake and DC activation [52]. Higher levels of expression of B7 molecules on infiltrating immune cells is consistent with increased stimulation of immune cells in tumors with higher T:S. Taken together, our data suggest that higher T:S is associated with greater activation of cross-presenting DCs, leading to greater activation of antigen-specific CD8^+^ T cells. Additionally, the concomitant higher levels of inhibitory molecule expression by intratumoral APCs and higher PD-1 expression by T cells suggest that T cells may be actively receiving inhibitory stimuli and therefore may play a role in responsiveness to PD-1/PD-L1 blockade, a model which has been supported by evidence from other groups [53–55]. Collectively, these data support a model where high stromal content may restrict immune cell activation. As a result, combining immunotherapies with drugs targeting the tumor stroma would increase responses rates.

### Immunological similarities and differences between metastatic tumor sites

Previous groups have noted immune heterogeneity between metastatic tumor sites in ovarian cancer [56–58]; however, the immune cell phenotype at the protein level has not been examined in detail. We have analyzed pairs of tumors from different metastatic sites of the same patient (*n* = 5) using our flow cytometry panels to characterize and compare the immune cell phenotypes between the two metastatic locations. We found the expression of certain markers was consistent between tumor sites. PD-1 expression on CD4^+^ T cells correlated strongly between tumors from the same patient but PD-1 expression on CD8^+^ T cells did not. It is possible that CD8^+^ T cells could be activated and expand differentially within the tumor depending on the TME and specific antigens present. On APCs, we found that expression of some markers such as PD-L1 and CD86 were strongly correlated between metastatic locations. Other markers such as CD40 and ICOSL were not significantly correlated. For other parameters such as the proportion of tumor cells in a tumor and the proportion of tumor cells expressing B7-H4, data from four of the five patients appeared to correlate strongly and one was an outlier.

Comparison of tumors from omentum or ovary revealed that omental tumors had greater frequencies of T and B lymphocytes, but the immune cell phenotype did not differ between metastatic sites (Additional file 2: Fig. S11). It is possible that the difference in cell number arises from populations of lymphocytes present in the milky spots of the omentum prior to tumor metastasis whereas lymphocytes present in the ovarian tumors are more likely to have been recruited. Overall, these data demonstrate that there are phenotypic similarities between metastatic sites.

### Potential combinatorial power of B7-H3-targeting agents with other therapeutics

Nearly half of patients with high-grade serous ovarian cancer have mutations affecting homologous recombination repair pathways [59]. PARP inhibitors have been approved for the treatment of ovarian cancer [60] and have selective activity in patients with BRCA mutations [59]. We have information on the BRCA mutation status for 9 of the patients included in this study, all of whom were confirmed wildtype. The tumor content of tumors from these patients ranged from 11.9–96.7% tumor cells suggesting that T:S was not related to BRCA mutation status. The consistently high expression of B7-H3 suggests that its expression is not dependent on BRCA deficiency and that targeting this molecule could be effective for the majority of patients and will not be restricted to patients with homologous recombination deficiencies. Because BRCA mutations are associated with increased neoantigen frequency that can lead to enhanced visibility by the immune system, B7-H3-targeting agents may be selectively able to activate an anti-tumor immune response in patients with homologous recombination deficiencies. However, our data favors an interpretation that B7-H3-targeting agents such as antibody-drug conjugates or CAR T cells may be more effective than checkpoint blockade. Additionally, the high expression of B7-H3 on stromal cells will allow for the targeting of fibroblasts by these methods. Fibroblasts have been reported to have immunosuppressive activities [32], which is supported by these data. Thus, targeting the stroma in combination with immune-activating therapies may synergize to improve the efficacy of immunotherapy in ovarian cancer.

## Conclusions

Our study demonstrates that B7-H3 is expressed by many cells in the TME including immune cells, tumor cells, and stromal cells. Stromal cells expressed particularly high levels of B7-H3 and B7-H3 was strongly associated with expression of stroma-related genes. This association between B7-H3 expression and the stroma indicates that variation in stromal content of tumors needs to be accounted for when using bulk expression data. Further, we have found that a higher T:S is associated with higher proportions of CD8^+^ T cells expressing high levels of PD-1, and higher levels of PD-1 ligand expression by APCs. Additionally, we found that B7-H4 expression was generally associated with tumor cells whereas PD-L1 was primarily expressed by stromal cells, resulting in a mutually exclusive pattern of expression. This work provides important insight into the expression patterns of members of the B7 family and furthers our understanding of the immune infiltrate in EOC.

## Supplementary information

**Additional file 1 Table S1:** Antibodies used for flow cytometry staining of tumor single cell suspensions. **Table S2:** Clinicopathologic information for ovarian cancer patients whose tumor specimens were analyzed by flow cytometry or immunohistochemistry.

**Additional file 2 Fig. S1:** B7-H3 immunohistochemistry of noncancerous human placenta, tonsil, spleen, and liver tissues. FFPE sections from various tissues were stained for B7-H3 expression by IHC. **Fig. S2:** B7-H3 and B7-H4 show different expression patterns on tumor and stromal cells in EOC. A: Flow cytometric staining of B7-H3 and B7-H4 on CD45^−^ cells can define tumor and stromal populations. B, C: Levels of B7-H3 (B) and B7-H4 (C) expression on tumor (red) and stromal (blue) cells. D: Levels of HLA-ABC expression on tumor (red) and stromal (blue) cells in relation to B7-H3 (top) and B7-H4 (bottom). Matched FMO control shown in grey (B,C,D). Examples shown are from three patients’ tumors. **Fig. S3:** Top 50 genes whose mRNA expression most significantly positively correlates with *CD276* mRNA expression across 22 TCGA datasets. Red text denotes genes with possible roles in the synthesis or modification of the ECM. **Fig. S4:** Gating schema for CD45^−^ population positive for epithelial or stromal markers. B7-H3 gMFI was calculated for viable, CD45^−^ singlets positive for epithelial (EpCAM, E-Cadherin) or stromal (FAP, PDGFRβ, PDPN, CD10) markers. **Fig. S5:** Comparative levels of B7-H3 expression between different tumor and stromal populations. A,B: Comparisons between levels of B7-H3 expression on FAP^high^ (A) or PDGFRβ^+^ (B) stromal cell populations and EpCAM^+^ tumor cells. C: Comparisons between proportions of FAP^high^ and PDGFRβ^+^ CD45^−^ cells. D: Proportions of total FAP^high^ with PDGFRβ^+^FAP^high^ cells in EOC samples. Points from the same patient are connected by a line. Significance was determined by paired T test. **Fig. S6:** Example of methods used to quantify tumor and stromal content of tumors. A: H&E stained slides categorized into tumor (red), stroma (green), and excluded (yellow) areas using HALO software. B: Flow plot of B7-H3 staining used to gate tumor (B7-H3^low^) and stromal (B7-H3^high^) cells. **Fig. S7:** Recurrence-free and overall survival in association with low or high tumor-to-stroma ratio (T:S). Recurrence-free (*p* = 0.098) and overall (*p* = 0.26) survival curves in patients with tumors with low (T:S < 1.5; blue) or high (T:S > 1.5; orange) T:S. Significance was determined by Mantel-Cox text. **Fig. S8:** Differences in immune cell phenotype is not due to NACT treatment. A,B,C: Comparison between frequency of PD-1^high^ CD4^+^ and CD8^+^ T cells (A), CD16 expression on monocytes (B), and activating (ICOSL, B7-H3, CD40, CD86, CLEC9a) and inhibitory (B7-H4, B7-H3, PD-L1, PD-L2) expression on APCs (C) from patients who did (circle) or did not (open square) receive NACT treatment within 6 months prior to surgery. Significance was determined by Mann Whitney U test. **Fig. S9:** T:S is not associated with differences in expression of exhaustion or activation markers by infiltrating T cells. A: Gating schema for determining coexpression of high levels of PD-1 with TIM3 and/or LAG3 on CD4^+^ and CD8^+^ T cells. B: Proportion of PD-1^high^ CD4^+^ (top) or CD8^+^ (bottom) T cells coexpressing TIM3, LAG3, or both. C,D: Expression of ICOS, TIA-1, GzmB, 2B4, CD107a, and GITR staining on CD4^+^ and CD8^+^ T cells directly *ex vivo*. Whisker and box plots summarize marker expression from tumors with low (< 1.5; blue) or high (> 1.5; orange) T:S. Statistical significance was determined by Mann Whitney U test (B,D). **Fig. S10:** Expression of activating, inhibitory, and maturation markers on B cells. A: Example stains of CD19^+^ B cells coexpressing CD20^+^CD22^+^, IgD^+^, BTLA^+^, or CD27^+^. B: Column plots showing marker expression on B cells from tumors with low (blue) or high (orange) T:S. Statistical significance was determined by Mann Whitney U test. **Fig. S11:** Immune cell frequency, but not phenotype, is associated with tumor location. A-D: Frequency of T cells (A), B cells (B), monocytes (C), and CD4^+^ and CD8^+^ T cells (D) isolated from ovary or omental tumors. E: Frequency of high levels of PD-1 expression on CD4^+^ or CD8^+^ T cells isolated from ovary or omental tumors. F: Level of CD16 expression on monocytes isolated from ovary or omental tumors. G: Level of expression of activating (ICOSL, B7-H3, CD40, CD86, CLEC9a) or inhibitory (B7-H3, B7-H4, PD-L1, PD-L2) markers on APCs isolated from ovary or omental tumors. Data from ovary tumors is shown in black, data from omental tumors is shown in purple. Statistical significance was determined by Mann Whitney U test.

## Data Availability

The TCGA datasets analyzed in this study are available through cBioPortal for Cancer Genomics.

## Abbreviations

APC: Antigen presenting cell
CAF: Cancer-associated fibroblast
ECM: Extracellular matrix
EOC: Epithelial ovarian cancer
FAP: Fibroblast activation protein alpha
FMO: Fluorescence minus one control
IHC: Immunohistochemistry
PDPN: Podoplanin
T:S: Tumor-to-stroma ratio
TME: Tumor microenvironment

## References

[1] Baumeister SH, Freeman GJ, Dranoff G, Sharpe AH (2016). Coinhibitory pathways in immunotherapy for Cancer. Annu Rev Immunol.

[2] Callahan Margaret K., Postow Michael A., Wolchok Jedd D. (2016). Targeting T Cell Co-receptors for Cancer Therapy. Immunity.

[3] Schildberg FA, Klein SR, Freeman GJ, Sharpe AH (2016). Coinhibitory pathways in the B7-CD28 ligand-receptor family. Immunity.

[4] Selby MJ, Engelhardt JJ, Quigley M, Henning KA, Chen T, Srinivasan M (2013). Anti-CTLA-4 antibodies of IgG2a Isotype enhance antitumor activity through reduction of Intratumoral regulatory T cells. Cancer Immunol Res.

[5] Simpson TR, Li F, Montalvo-Ortiz W, Sepulveda MA, Bergerhoff K, Arce F (2013). Fc-dependent depletion of tumor-infiltrating regulatory T cells co-defines the efficacy of anti–CTLA-4 therapy against melanoma. J Exp Med.

[6] Bowtell DD, Böhm S, Ahmed AA, Aspuria P-J, Bast RC, Beral V (2015). Rethinking ovarian cancer II: reducing mortality from high-grade serous ovarian cancer. Nat Rev Cancer.

[7] Zhang L, Conejo-Garcia JR, Katsaros D, Gimotty PA, Massobrio M, Regnani G (2003). Intratumoral T cells, recurrence, and survival in epithelial ovarian Cancer. N Engl J Med.

[8] Nielsen JS, Sahota RA, Milne K, Kost SE, Nesslinger NJ, Watson PH (2012). CD20+ tumor-infiltrating lymphocytes have an atypical CD27- memory phenotype and together with CD8+ T cells promote favorable prognosis in ovarian Cancer. Clin Cancer Res.

[9] Hamanishi J, Mandai M, Ikeda T, Minami M, Kawaguchi A, Murayama T (2015). Safety and antitumor activity of anti-PD-1 antibody, nivolumab, in patients with platinum-resistant ovarian cancer. J Clin Oncol.

[10] Disis ML, Patel MR, Pant S, Infante JR, Lockhart AC, Kelly K (2015). Avelumab (MSB0010718C), an anti-PD-L1 antibody, in patients with previously treated, recurrent or refractory ovarian cancer: a phase Ib, open-label expansion trial. J Clin Oncol.

[11] Varga A, Piha-Paul SA, Ott PA, Mehnert JM, Berton-Rigaud D, Johnson EA (2015). Antitumor activity and safety of pembrolizumab in patients (pts) with PD-L1 positive advanced ovarian cancer: interim results from a phase Ib study. J Clin Oncol.

[12] Clouthier DL, Lien SC, Yang SYC, Nguyen LT, Manem VSK, Gray D (2019). An interim report on the investigator-initiated phase 2 study of pembrolizumab immunological response evaluation ( INSPIRE ). J Immunother Cancer.

[13] Podojil Joseph R., Chiang Ming-Yi, Ifergan Igal, Copeland Ronald, Liu Linda N., Maloveste Sebastien, Langermann Solomon, Liebenson David, Balabanov Roumen, Chi Hongbo, Chen Lieping, Vignali Dario A. A., Miller Stephen D. (2018). B7-H4 Modulates Regulatory CD4+ T Cell Induction and Function via Ligation of a Semaphorin 3a/Plexin A4/Neuropilin-1 Complex. The Journal of Immunology.

[14] Ohaegbulam KC, Liu W, Jeon H, Almo SC, Zang X (2017). Tumor-expressed immune checkpoint B7x promotes cancer progression and antigen-specific CD8 T cell exhaustion and suppressive innate immune cells. Oncotarget.

[15] MacGregor HL, Ohashi PS (2017). Molecular pathways: evaluating the potential for B7-H4 as an Immunoregulatory target. Clin Cancer Res.

[16] Picarda E, Ohaegbulam KC, Zang X (2016). Molecular pathways: targeting B7-H3 (CD276) for human cancer immunotherapy. Clin Cancer Res.

[17] Zhang X, Fang C, Zhang G, Jiang F, Wang L, Hou J (2017). Prognostic value of B7-H3 expression in patients with solid tumors: a meta-analysis. Oncotarget.

[18] Mak MP, Tong P, Diao L, Cardnell RJ, Gibbons DL, William WN (2015). A patient-derived, pan-Cancer EMT signature identifies global molecular alterations and immune target enrichment following epithelial-to-Mesenchymal transition. Clin Cancer Res.

[19] Li Y, Yang X, Wu Y, Zhao K, Ye Z, Zhu J (2017). B7-H3 promotes gastric cancer cell migration and invasion. Oncotarget.

[20] Zhang C, Zhang Z, Li F, Shen Z, Qiao Y, Li L (2018). Large-scale analysis reveals the specific clinical and immune features of B7-H3 in glioma. Oncoimmunology.

[21] Lemke D, Pfenning P-N, Sahm F, Klein A-C, Kempf T, Warnken U (2012). Costimulatory protein 4IgB7H3 drives the malignant phenotype of glioblastoma by mediating immune escape and invasiveness. Clin Cancer Res.

[22] Zang X, Sullivan PS, Soslow RA, Waitz R, Reuter VE, Wilton A (2010). Tumor associated endothelial expression of B7-H3 predicts survival in ovarian carcinomas. Mod Pathol.

[23] Ingebrigtsen VA, Boye K, Tekle C, Nesland JM, Flatmark K, Fodstad O (2012). B7-H3 expression in colorectal cancer: nuclear localization strongly predicts poor outcome in colon cancer. Int J Cancer.

[24] Brunner A, Hinterholzer S, Riss P, Heinze G, Brustmann H (2012). Immunoexpression of B7-H3 in endometrial cancer: relation to tumor T-cell infiltration and prognosis. Gynecol Oncol.

[25] Crispen PL, Sheinin Y, Roth TJ, Lohse CM, Kuntz SM, Frigola X (2008). Tumor cell and tumor vasculature expression of B7-H3 predict survival in clear cell renal cell carcinoma. Clin Cancer Res.

[26] Costa A, Kieffer Y, Scholer-Dahirel A, Pelon F, Bourachot B, Cardon M (2018). Fibroblast heterogeneity and immunosuppressive environment in human breast Cancer. Cancer Cell.

[27] Gao J., Aksoy B. A., Dogrusoz U., Dresdner G., Gross B., Sumer S. O., Sun Y., Jacobsen A., Sinha R., Larsson E., Cerami E., Sander C., Schultz N. (2013). Integrative Analysis of Complex Cancer Genomics and Clinical Profiles Using the cBioPortal. Science Signaling.

[28] Cerami E, Gao J, Dogrusoz U, Gross BE, Sumer SO, Aksoy BA (2012). The cBio Cancer genomics portal: an open platform for exploring multidimensional cancer genomics data. Cancer Discov.

[29] Petroff MG, Kharatyan E, Torry DS, Holets L (2005). The Immunomodulatory proteins B7-DC, B7-H2, and B7-H3 are differentially expressed across gestation in the human placenta. Am J Pathol.

[30] Shi L, Chen L, Wu C, Zhu Y, Xu B, Zheng X (2016). PD-1 blockade boosts radiofrequency ablation-elicited adaptive immune responses against tumor. Clin Cancer Res.

[31] Liu Zixing, Zhang Wenling, Phillips Joshua B., Arora Ritu, McClellan Steven, Li Jiangfeng, Kim Jin-Hwan, Sobol Robert W., Tan Ming (2018). Immunoregulatory protein B7-H3 regulates cancer stem cell enrichment and drug resistance through MVP-mediated MEK activation. Oncogene.

[32] Kalluri R (2016). The biology and function of fibroblasts in cancer. Nat Rev Cancer.

[33] Wu J, Liang C, Chen M, Su W (2016). Association between tumor-stroma ratio and prognosis in solid tumor patients: a systematic review and meta-analysis. Oncotarget.

[34] Smith JB, Lanitis E, Dangaj D, Buza E, Poussin M, Stashwick C (2016). Tumor regression and delayed onset toxicity following B7-H4 CAR T cell therapy. Mol Ther.

[35] Hansen JM, Coleman RL, Sood AK (2017). Targeting the tumor microenvironment in ovarian Cancer. Eur J Cancer.

[36] Mariathasan S, Turley SJ, Nickles D, Castiglioni A, Yuen K, Wang Y (2018). TGFβ attenuates tumour response to PD-L1 blockade by contributing to exclusion of T cells. Nature.

[37] Altan M, Pelekanou V, Schalper KA, Toki M, Gaule P, Syrigos K (2017). B7-H3 expression in NSCLC and its association with B7-H4, PD-L1 and tumor-infiltrating lymphocytes. Clin Cancer Res.

[38] Finak G, Bertos N, Pepin F, Sadekova S, Souleimanova M, Zhao H (2008). Stromal gene expression predicts clinical outcome in breast cancer. Nat Med.

[39] Chen GM, Kannan L, Geistlinger L, Kofia V, Safikhani Z, Gendoo DMA (2018). Consensus on molecular subtypes of high-grade serous ovarian carcinoma. Clin Cancer Res.

[40] Farmer P, Bonnefoi H, Anderle P, Cameron D, Wirapati P, Becette V (2009). A stroma-related gene signature predicts resistance to neoadjuvant chemotherapy in breast cancer. Nat Med.

[41] Saleh MN, Goldman SJ, LoBuglio AF, Beall AC, Sabio H, McCord MC (1995). CD16+ monocytes in patients with Cancer: spontaneous elevation and pharmacologic induction by recombinant human macrophage Colony-stimulating factor. Blood.

[42] Romano E, Kusio-Kobialka M, Foukas PG, Baumgaertner P, Meyer C, Ballabeni P (2015). Ipilimumab-dependent cell-mediated cytotoxicity of regulatory T cells ex vivo by nonclassical monocytes in melanoma patients. Proc Natl Acad Sci U S A.

[43] Phillips JH, Chang C, Lanier LL (1991). Platelet-induced expression of Fcy RIII (CD16) on human monocytes. Eur J Immunol.

[44] Al-Rashed F, Ahmad Z, Iskandar MA, Tuomilehto J, Al-Mulla F, Ahmad R (2019). TNF-α induces a pro-inflammatory phenotypic shift in monocytes through ACSL1: relevance to metabolic inflammation. Cell Physiol Biochem.

[45] Victor AR, Weigel C, Scoville SD, Chan WK, Chatman K, Nemer MM (2018). Epigenetic and posttranscriptional regulation of CD16 expression during human NK cell development. J Immunol.

[46] Xu H, Sun Q, Lu L, Luo F, Zhou L, Liu J (2017). MicroRNA-218 acts by repressing TNFR1-mediated activation of NF-κB, which is involved in MUC5AC hyper-production and inflammation in smoking-induced bronchiolitis of COPD. Toxicol Lett.

[47] Antonelli LRV, Leoratti FMS, Costa PAC, Rocha BC, Diniz SQ, Tada MS (2014). The CD14+CD16+ inflammatory monocyte subset displays increased mitochondrial activity and effector function during acute plasmodium vivax malaria. PLoS Pathog.

[48] Belge K-U, Dayyani F, Horelt A, Siedlar M, Frankenberger M, Frankenberger B (2002). The Proinflammatory CD14+CD16+DR++ monocytes are a major source of TNF. J Immunol.

[49] Romee R, Foley B, Lenvik T, Wang Y, Zhang B, Ankarlo D (2013). NK cell CD16 surface expression and function is regulated by a disintegrin and metalloprotease-17 (ADAM17). Blood.

[50] Miller MA, Sullivan RJ, Lauffenburger DA (2017). Molecular pathways: receptor ectodomain shedding in treatment, resistance, and monitoring of cancer. Clin Cancer Res.

[51] Sancho D, Mourão-Sá D, Joffre OP, Schulz O, Rogers NC, Pennington DJ (2008). Tumor therapy in mice via antigen targeting to a novel, DC-restricted C-type lectin. J Clin Invest.

[52] Schreibelt G, Klinkenberg LJJ, Cruz LJ, Tacken PJ, Tel J, Kreutz M (2012). The C-type lectin receptor CLEC9A mediates antigen uptake and (cross-)presentation by human blood BDCA3+ myeloid dendritic cells. Blood.

[53] Herbst RS, Soria JC, Kowanetz M, Fine GD, Hamid O, Gordon MS (2014). Predictive correlates of response to the anti-PD-L1 antibody MPDL3280A in cancer patients. Nature.

[54] Lin H, Wei S, Hurt EM, Green MD, Zhao L, Vatan L (2018). Host expression of PD-L1 determines efficacy of PD-L1 pathway blockade–mediated tumor regression. J Clin Invest.

[55] Flies DB, Higuchi T, Harris JC, Jha V, Gimotty PA, Adams SF (2016). Immune checkpoint blockade reveals the stimulatory capacity of tumor-associated CD103+ dendritic cells in late-stage ovarian cancer. Oncoimmunology.

[56] Hagemann AR, Hagemann IS, Cadungog M, Hwang WT, Patel P, Lal P (2011). Tissue-based immune monitoring II: multiple tumor sites reveal immunologic homogeneity in serous ovarian carcinoma. Cancer Biol Ther.

[57] Dötzer K, Schlüter F, Schoenberg MB, Bazhin AV, von Koch FE, Schnelzer A (2019). Immune heterogeneity between primary tumors and corresponding metastatic lesions and response to platinum therapy in primary ovarian Cancer. Cancers (Basel).

[58] Zhang AW, Mcpherson A, Milne K, Kroeger DR, Hamilton PT, Miranda A, et al. Interfaces of malignant and immunologic clonal dynamics in ovarian cancer. Cell. 2018;173:1755-69. 10.1016/j.cell.2018.03.073 29754820

[59] Neff RT, Senter L, Salani R (2017). BRCA mutation in ovarian cancer: testing, implications and treatment considerations. Ther Adv Med Oncol.

[60] Moore KN, Raza M, Matulonis UA (2018). The poly (ADP ribose) polymerase inhibitor niraparib: management of toxicities. Gynecol Oncol.

