# Supplementary material for: High expression of B7-H3 on stromal cells defines tumor and stromal compartments in epithelial ovarian cancer and is associated with limited immune activation
Source: J Immunother Cancer. 2019 Dec 31;7:357. doi: 10.1186/s40425-019-0816-5 (PMC6937725; doi:10.1186/s40425-019-0816-5)
Supplement: Supplementary file 1 — Additional file 1 Table S1: Antibodies used for flow cytometry staining of tumor single cell suspensions. Table S2: Clinicopathologic information for ovarian cancer patients whose tumor specimens were analyzed by flow cytometry or immunohistochemistry. [file 40425_2019_816_MOESM1_ESM.docx]

Supplementary Table 1: Antibodies used for flow cytometry staining of tumor single cell suspensions.

| **Antigen** | **Antibody clone** | **Supplier** | **Catalog number** | **Fluorophore** |
| --- | --- | --- | --- | --- |
| B7-H3 | MIH42 | BioLegend | 351007 | PE/Cy7 |
| B7-H3 | 185504 | R&D Systems | FAB1027P | PE |
| B7-H4 | MIH43 | GeneTex | GTX43788 | FITC |
| FAP | 427819 | R&D Systems | FAB3715P | PE |
| CD10 | HI10a | BD Biosciences | 563509 | AF700 |
| PDPN | NC-08 | BioLegend | 337011 | PerCP/Cy5.5 |
| PDGFRβ | 28D4 | BD Biosciences | 564124 | BV421 |
| EpCAM | 9C4 | BioLegend | 324237 | BV785 |
| E-Cadherin | 67A4 | BD Biosciences | 563572 | PE-CF594 |
| CD45 | H130 | BioLegend | 304005 | FITC |
| CD3 | UCHT1 | ThermoFisher | 25-0038-41 | PE/Cy7 |
| CD19 | SJ25C1 | BD Biosciences | 557835 | PE/Cy7 |
| CD14 | M5E2 | BD Biosciences | 558121 | Pacific Blue |
| CD16 | 3G8 | Biolegend | 302017 | APC/Cy7 |
| CD11c | 3.9 | BioLegend | 301625 | Pacific Blue |
| HLA-DR | G46-6 | BD Biosciences | 560652 | PerCP/Cy5.5 |
| ICOS-L | 2D3 | BioLegend | 309403 | PE |
| PD-L1 | MIH1 | BD Biosciences | 558017 | PE/Cy7 |
| PD-L2 | MIH18 | BD Biosciences | 557926 | APC |
| CD40 | 5C3 | BioLegend | 334323 | APC/Cy7 |
| CD86 | IT2.2 | BioLegend | 305421 | PE/Cy7 |
| CLEC9a | 683409 | R&D Systems | FAB6049A | APC |
| CD4 | RPA-T4 | BD Biosciences | 557871 | APC/Cy7 |
| CD8 | RPA-T8 | BD Biosciences | 558207 | Pacific Blue |
| PD-1 | eBioJ105 | ThermoFisher | 46-2799-41 | PercCP-eFluor710 |
| TIM3 | 344823 | R&D Systems | FAB2365P | PE |
| LAG3 | 17B4 | Enzo | ALX-804-806 | FITC |
| CD107a | H4A3 | BD Biosciences | 555801 | PE |
| 2B4 | C1.7 | BioLegend | 329515 | PerCP/Cy5.5 |
| GITR | eBioAITR | ThermoFisher | 25-5875-41 | PE/Cy7 |
| ICOS | 398.4A | BioLegend | 313514 | AF488 |
| TIA-1 | 2G9A10F5 | Beckman Coulter | IM3293 | PE |
| GzmB | GB11 | BD Biosciences | 561142 | PE |

Supplementary Table 2: Clinicopathologic information for ovarian cancer patients whose tumor specimens were analyzed by flow cytometry or immunohistochemistry.

| **OV#** | **Age at diagnosis** | **Histological type (from final path report)** | **Histological grade: WHO Grading System (Two Tier System)** | **Primary or recurrent disease** | **Neoadjuvant chemotherapy (Y/N)** | **Debulking status** | **Platinum resistance** |
| --- | --- | --- | --- | --- | --- | --- | --- |
| OV370 | 74 | high grade serous carcinoma | G3 | primary | Y | optimal (residual disease) | resistant |
| OV374 | 71 | high grade serous carcinoma | G3 (high grade) | primary | Y | optimal (residual disease) | sensitive |
| OV381 | 68 | high grade serous carcinoma | G3 | primary | Y | optimal | resistant |
| OV392 | 70 | high grade serous carcinoma | G3 (high grade) | primary | N | suboptimal | resistant |
| OV397 | 67 | high grade serous carcinoma | GX (high grade) | primary | Y | suboptimal | resistant |
| OV403 | 51 | high grade serous carcinoma | GX (high grade) | primary | N | optimal (residual disease) | sensitive |
| OV413 | 74 | high grade serous carcinoma | GX (high grade) | primary | Y | optimal | sensitive |
| OV414 | 69 | high grade serous carcinoma | G3 | primary | Y | suboptimal | resistant |
| OV438 | 42 | high grade serous carcinoma | GX (high grade) | primary | N | optimal (residual disease) | sensitive |
| OV439 | 60 | high grade serous carcinoma | G3 | primary | N | optimal (residual disease) | sensitive |
| OV476 | 65 | high grade serous carcinoma | n/a | primary | Y | suboptimal | resistant |
| OV484 | 79 | high grade serous carcinoma | G3 | primary | Y | optimal (residual disease) | sensitive |
| OV496 | 73 | high grade serous carcinoma | GX (high grade) | primary | N | optimal (residual disease) | sensitive |
| OV557 | 53 | high grade serous carcinoma | G3 | primary | Y | optimal (residual disease) | resistant |
| OV558 | 47 | endometrioid | G2 | primary | N | optimal | no chemo |
| OV563 | 75 | endometrioid | G2 | primary | N | optimal | no chemo |
| OV567 | 60 | low grade serous carcinoma | not stated | recurrence | Y | optimal (residual disease) | no chemo |
| OV621 | 49 | high grade serous carcinoma | G3 | primary | N | optimal | no chemo |
| OV626 | 64 | high grade serous carcinoma | G3 | primary | Y | optimal | no chemo |
| OV627 | 68 | clear cell carcinoma | G3 | primary | N | suboptimal | no chemo |
| OV404 | 67 | sarcoma and high grade serous carcinoma | G3 | primary | N | not stated | sensitive |
| OV411 | 45 | high grade serous carcinoma | G3 | primary | N | optimal | resistant |
| OV425 | 76 | high grade serous carcinoma | G3 | primary | N | not stated | sensitive |
| OV536 | 53 | endometrioid | G3 | primary | Y | optimal | sensitive |
| OV560 | 57 | high grade serous carcinoma | G3 | primary | N | optimal | sensitive |
| OV571 | 47 | high grade serous carcinoma | G3 | primary | N | suboptimal | sensitive |
| OV587 | 69 | high grade serous carcinoma | not stated | recurrence | N | not stated | sensitive |
| OV180 | 81 | high grade serous carcinoma | n/a | recurrence | N (last 6 months) | n/a | sensitive |
| OV182 | 67 | high grade serous carcinoma | G3 | primary | Y | suboptimal | resistant |
| OV184 | 67 | high grade serous carcinoma | G3 | primary | Y | optimal | n/a |
| OV188 | 71 | high grade serous carcinoma | G3 | primary | N (last 6 months) | optimal | sensitive |
| OV194 | 82 | high grade serous carcinoma | G3 | primary | N | suboptimal | no chemo |
| OV263 | 52 | high grade serous carcinoma | G3 | recurrence | N (last 6 months) | n/a | sensitive |
| OV275/276 | 67 | clear cell carcinoma | GX | primary | Y | suboptimal | resistant |
