# Supplementary material for: High expression of B7-H3 on stromal cells defines tumor and stromal compartments in epithelial ovarian cancer and is associated with limited immune activation
Source: J Immunother Cancer. 2019 Dec 31;7:357. doi: 10.1186/s40425-019-0816-5 (PMC6937725; doi:10.1186/s40425-019-0816-5)
Supplement: Supplementary file 2 — Additional file 2 Fig. S1: B7-H3 immunohistochemistry of noncancerous human placenta, tonsil, spleen, and liver tissues. FFPE sections from various tissues were stained for B7-H3 expression by IHC. Fig. S2: B7-H3 and B7-H4 show different expression patterns on tumor and stromal cells in EOC. A: Flow cytometric staining of B7-H3 and B7-H4 on CD45− cells can define tumor and stromal populations. B, C: Levels of B7-H3 (B) and B7-H4 (C) expression on tumor (red) and stromal (blue) cells. D: Levels of HLA-ABC expression on tumor (red) and stromal (blue) cells in relation to B7-H3 (top) and B7-H4 (bottom). Matched FMO control shown in grey (B,C,D). Examples shown are from three patients’ tumors. Fig. S3: Top 50 genes whose mRNA expression most significantly positively correlates with CD276 mRNA expression across 22 TCGA datasets. Red text denotes genes with possible roles in the synthesis or modification of the ECM. Fig. S4: Gating schema for CD45− population positive for epithelial or stromal markers. B7-H3 gMFI was calculated for viable, CD45− singlets positive for epithelial (EpCAM, E-Cadherin) or stromal (FAP, PDGFRβ, PDPN, CD10) markers. Fig. S5: Comparative levels of B7-H3 expression between different tumor and stromal populations. A,B: Comparisons between levels of B7-H3 expression on FAPhigh (A) or PDGFRβ+ (B) stromal cell populations and EpCAM+ tumor cells. C: Comparisons between proportions of FAPhigh and PDGFRβ+ CD45− cells. D: Proportions of total FAPhigh with PDGFRβ+FAPhigh cells in EOC samples. Points from the same patient are connected by a line. Significance was determined by paired T test. Fig. S6: Example of methods used to quantify tumor and stromal content of tumors. A: H&E stained slides categorized into tumor (red), stroma (green), and excluded (yellow) areas using HALO software. B: Flow plot of B7-H3 staining used to gate tumor (B7-H3low) and stromal (B7-H3high) cells. Fig. S7: Recurrence-free and overall survival in association with low or high tum [file 40425_2019_816_MOESM2_ESM.pdf]

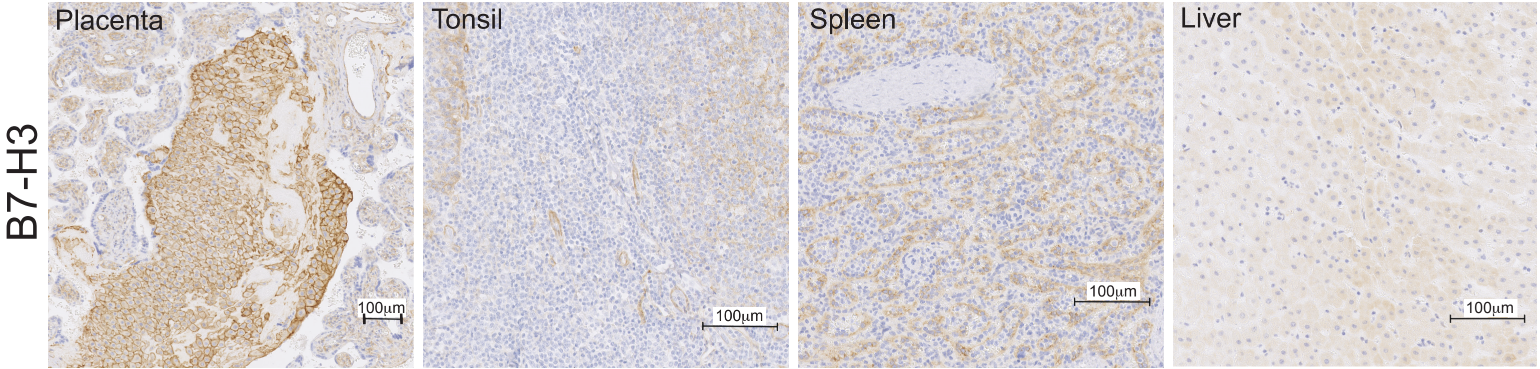
B7-H3
Placenta
Tonsil
Spleen
Liver
100μm
100μm
100μm
100μm

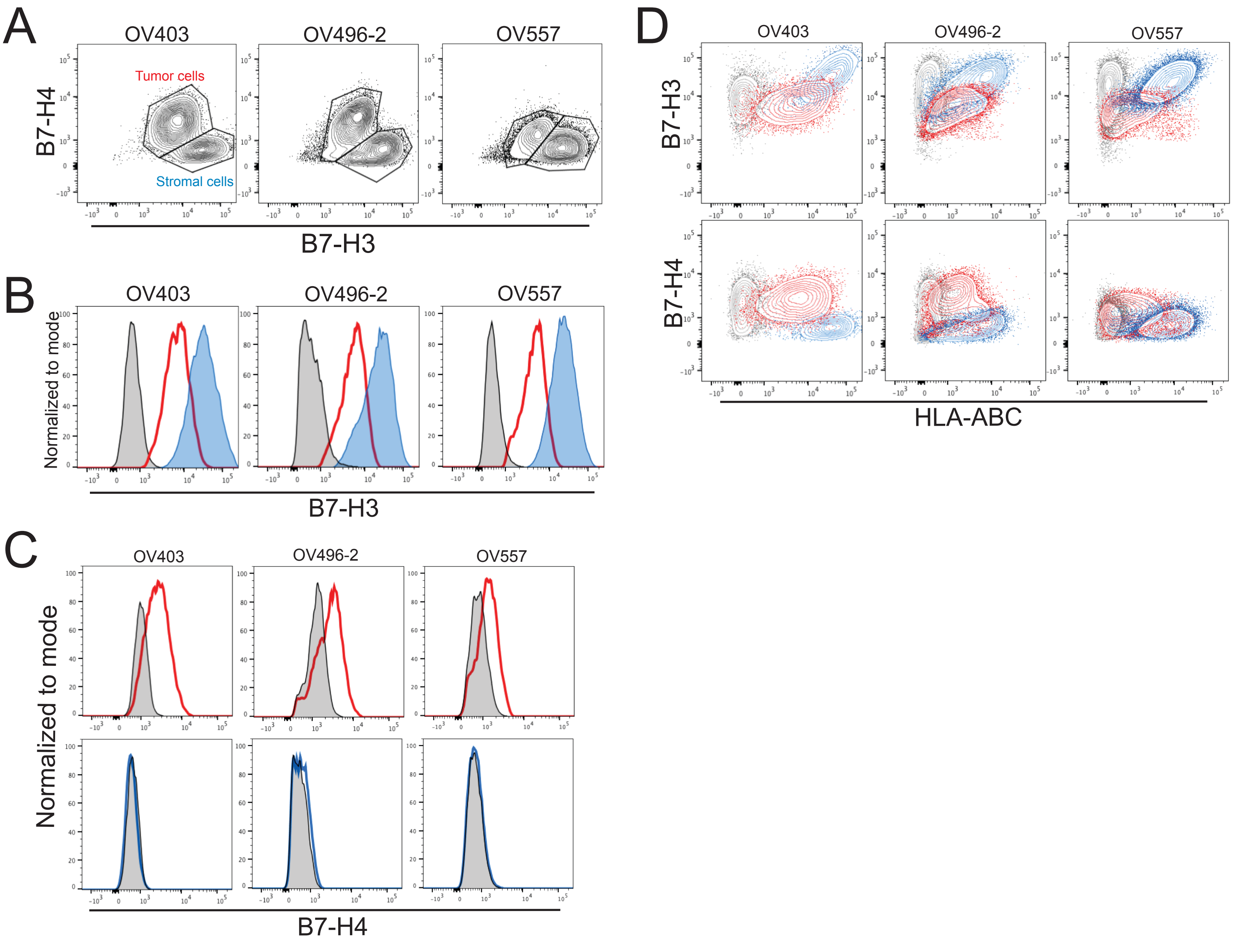

A
OV403
OV496-2
OV557
B7-H4
Tumor cells
Stromal cells
B7-H3
B
Normalized to mode
C
D
B7-H3
B7-H4
HLA-ABC

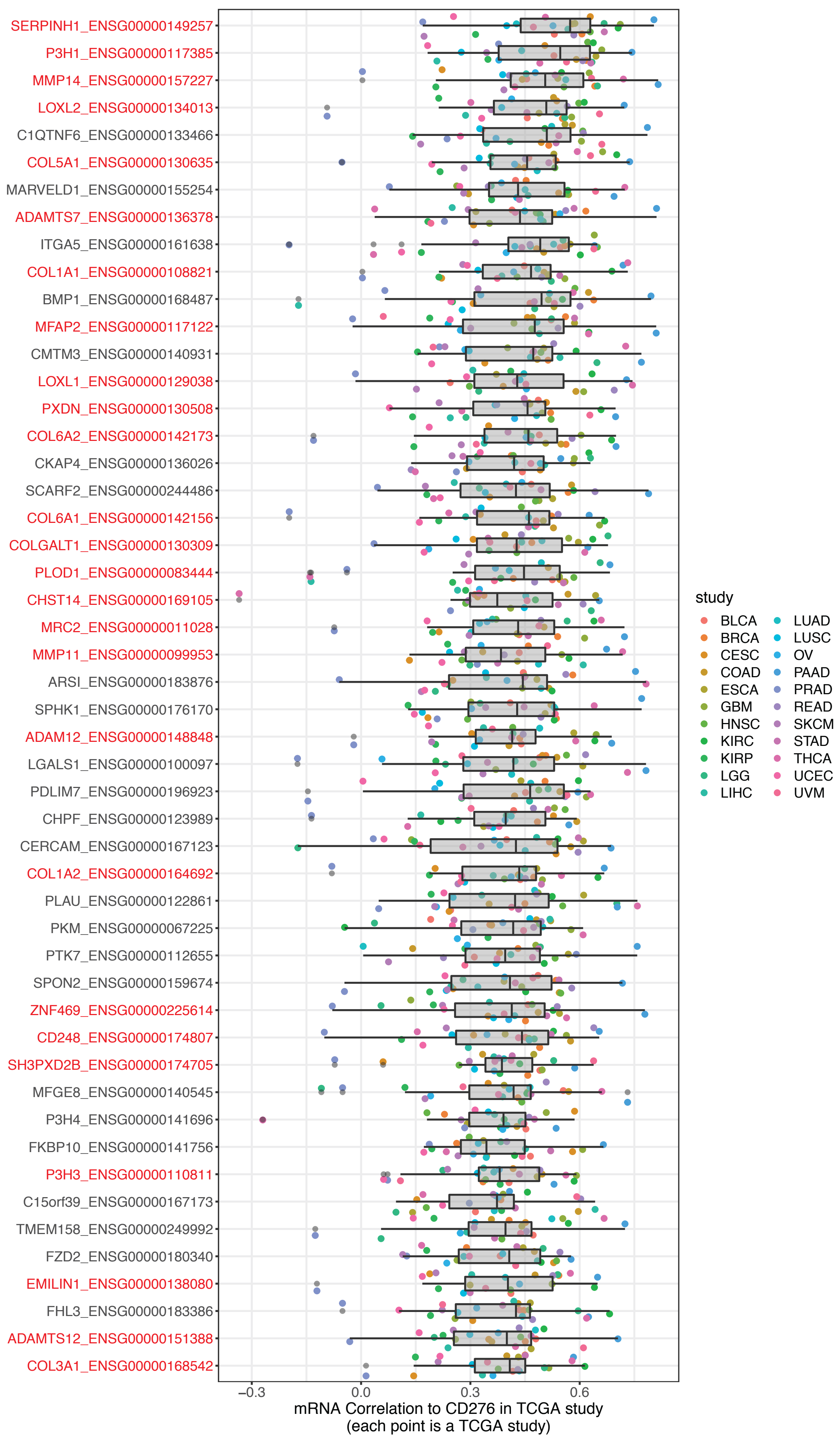
SERPINH1_ENSG00000149257
P3H1_ENSG00000117385
MMP14_ENSG00000157227
LOXL2_ENSG00000134013
C1QTNF6_ENSG00000133466
COL5A1_ENSG00000130635
MARVELD1_ENSG00000155254
ADAMTS7_ENSG00000136378
ITGA5_ENSG00000161638
COL1A1_ENSG00000108821
BMP1_ENSG00000168487
MFAP2_ENSG00000117122
CMTM3_ENSG00000140931
LOXL1_ENSG00000129038
PXDN_ENSG00000130508
COL6A2_ENSG00000142173
CKAP4_ENSG00000136026
SCARF2_ENSG00000244486
COL6A1_ENSG00000142156
COLGALT1_ENSG00000130309
PLOD1_ENSG00000083444
CHST14_ENSG00000169105
MRC2_ENSG00000011028
MMP11_ENSG00000099953
ARSI_ENSG00000183876
SPHK1_ENSG00000176170
ADAM12_ENSG00000148848
LGALS1_ENSG00000100097
PDLIM7_ENSG00000196923
CHPF_ENSG00000123989
CERCAM_ENSG00000167123
COL1A2_ENSG00000164692
PLAU_ENSG00000122861
PKM_ENSG00000067225
PTK7_ENSG00000112655
SPON2_ENSG00000159674
ZNF469_ENSG00000225614
CD248_ENSG00000174807
SH3PXD2B_ENSG00000174705
MFGE8_ENSG00000140545
P3H4_ENSG00000141696
FKBP10_ENSG00000141756
P3H3_ENSG00000110811
C15orf39_ENSG00000167173
TMEM158_ENSG00000249992
FZD2_ENSG00000180340
EMILIN1_ENSG00000138080
FHL3_ENSG00000183386
ADAMTS12_ENSG00000151388
COL3A1_ENSG00000168542
−0.3
0.0
0.3
0.6
mRNA Correlation to CD276 in TCGA study
(each point is a TCGA study)
study
BLCA
BRCA
CESC
COAD
ESCA
GBM
HNSC
KIRC
KIRP
LGG
LIHC
LUAD
LUSC
OV
PAAD
PRAD
READ
SKCM
STAD
THCA
UCEC
UVM

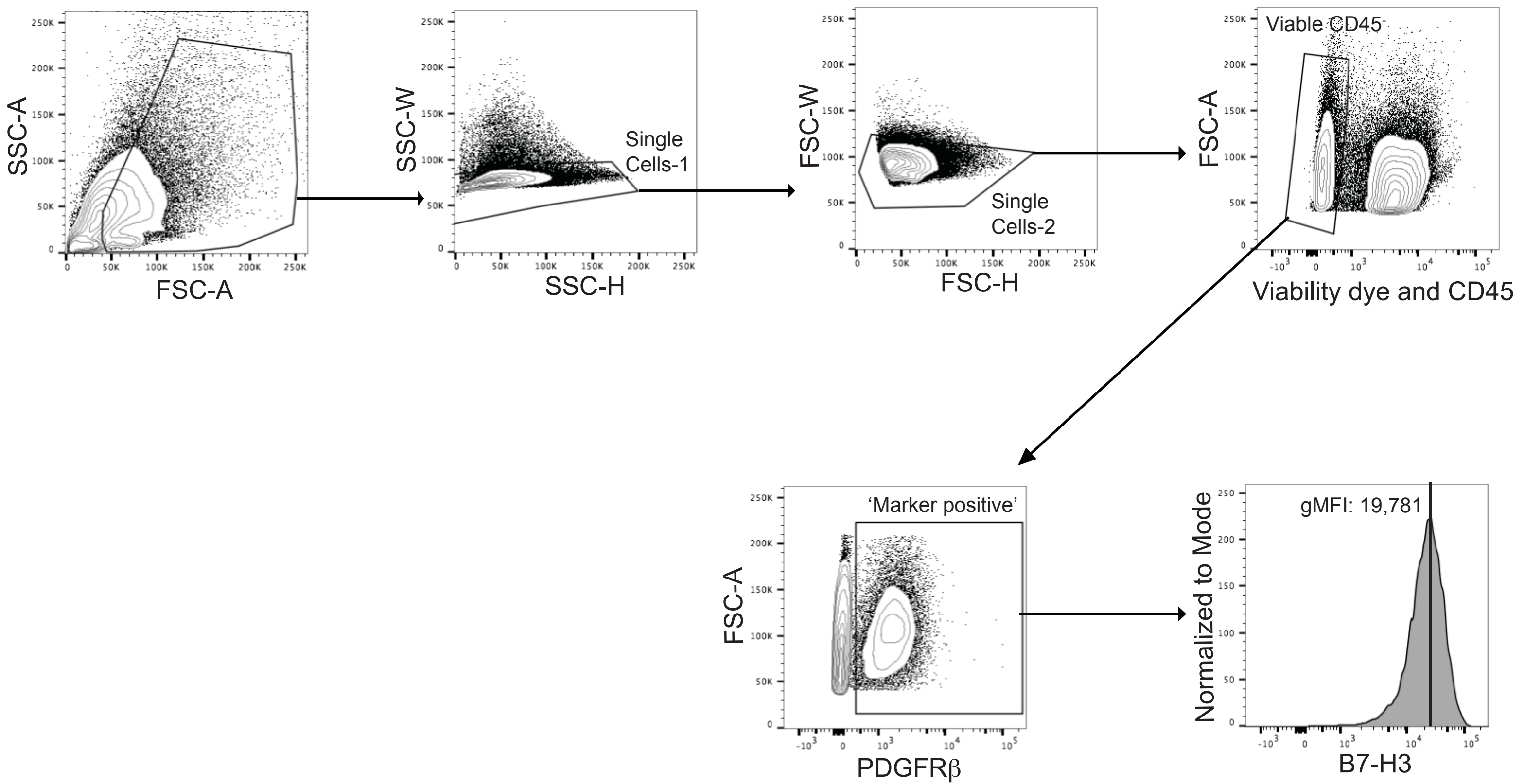

SSC-A
FSC-A
SSC-W
SSC-H
Single Cells-1
FSC-W
FSC-H
Single Cells-2
FSC-A
Viable CD45-
Viability dye and CD45
FSC-A
'Marker positive'
PDGFRβ
Normalized to Mode
gMFI: 19,781
B7-H3

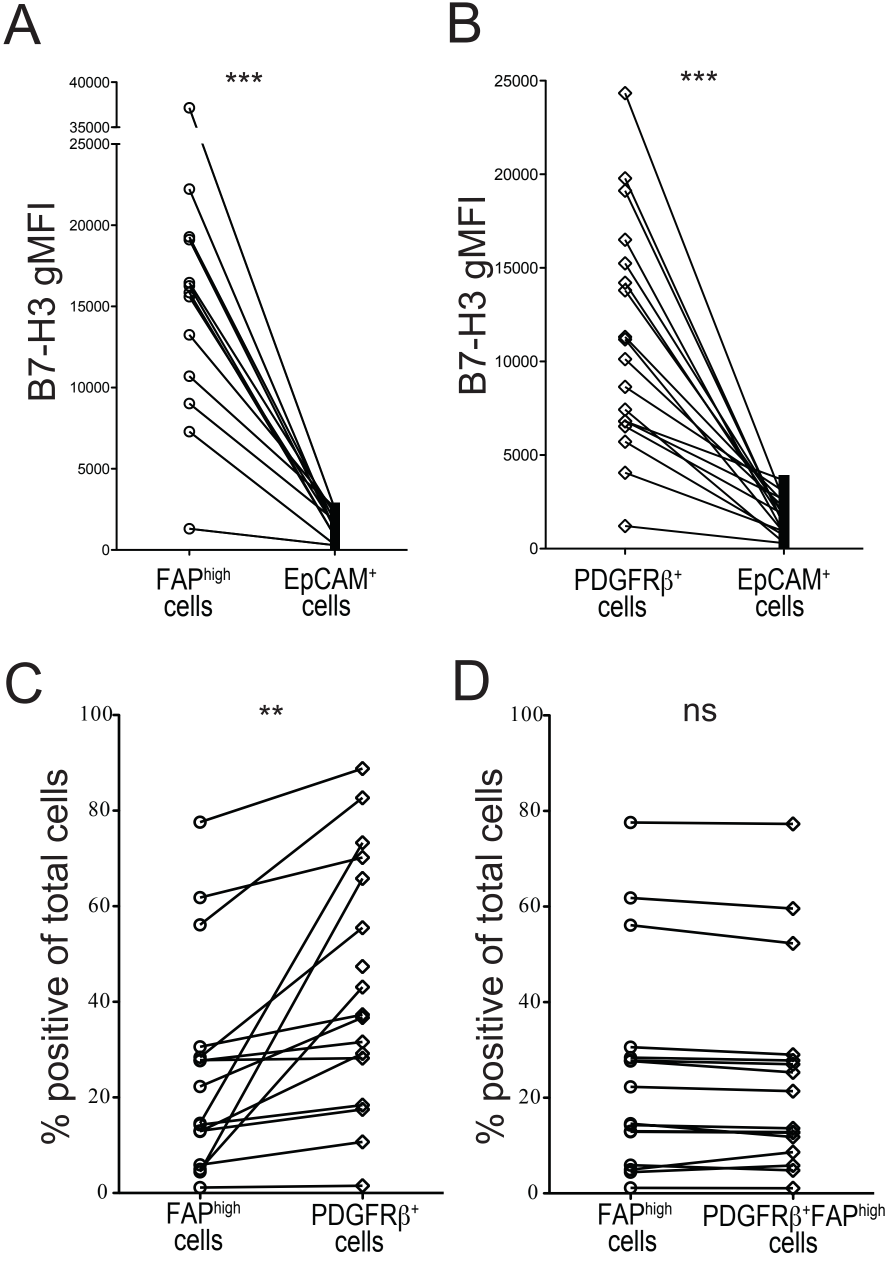

A
B
C
D
***
***
**
ns
B7-H3 gMFI
40000
35000
25000
20000
15000
10000
5000
0
FAPhigh cells
EpCAM+ cells
PDGFRβ+ cells
% positive of total cells
100
80
60
40
20
PDGFRβ+FAPhigh cells

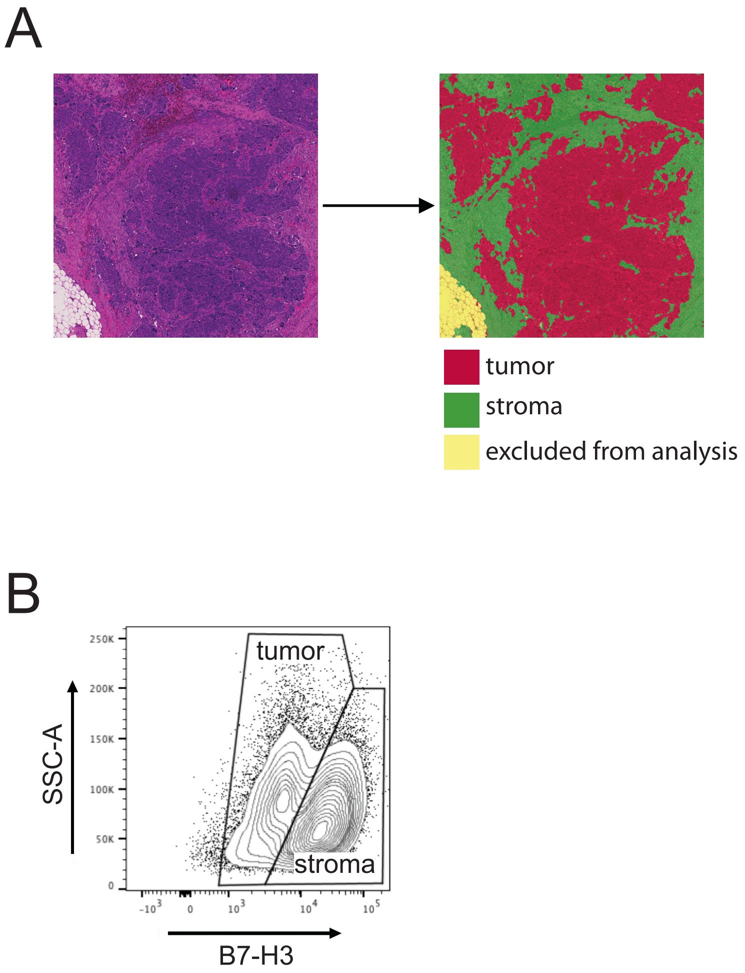
A
tumor
stroma
excluded from analysis
B
tumor
stroma
SSC-A
250K
200K
150K
100K
50K
0
-10^3
0
10^3
10^4
10^5
B7-H3

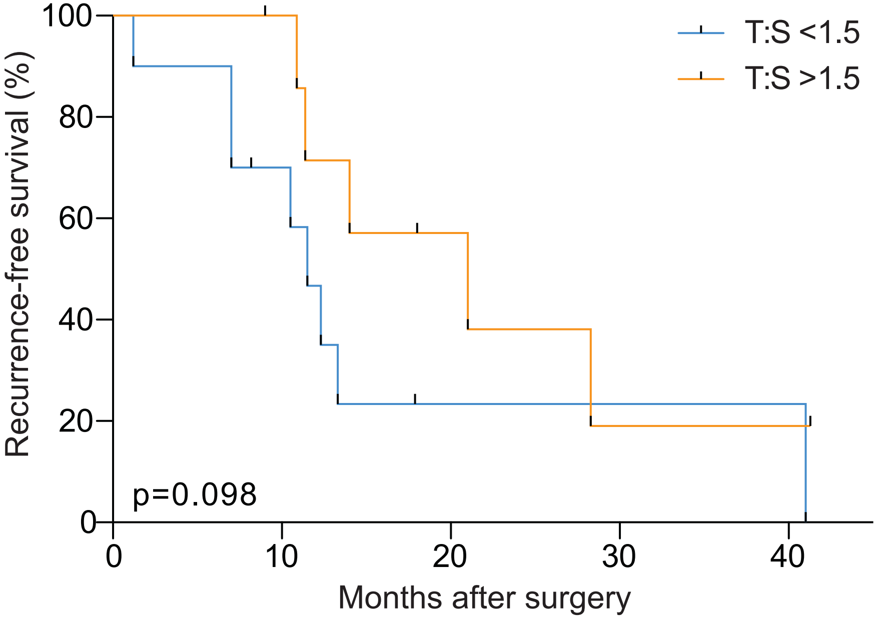

Recurrence-free survival (%)
100
80
60
40
20
0
T:S <1.5
T:S >1.5
p=0.098
0
10
20
30
40
Months after surgery

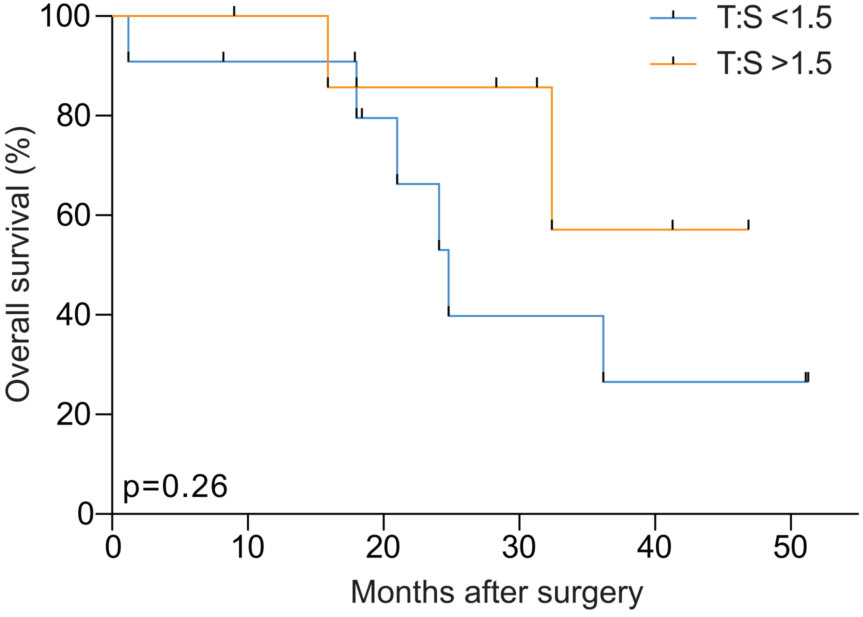

Overall survival (%)
100
80
60
40
20
0
T:S <1.5
T:S >1.5
p=0.26
0
10
20
30
40
50
Months after surgery

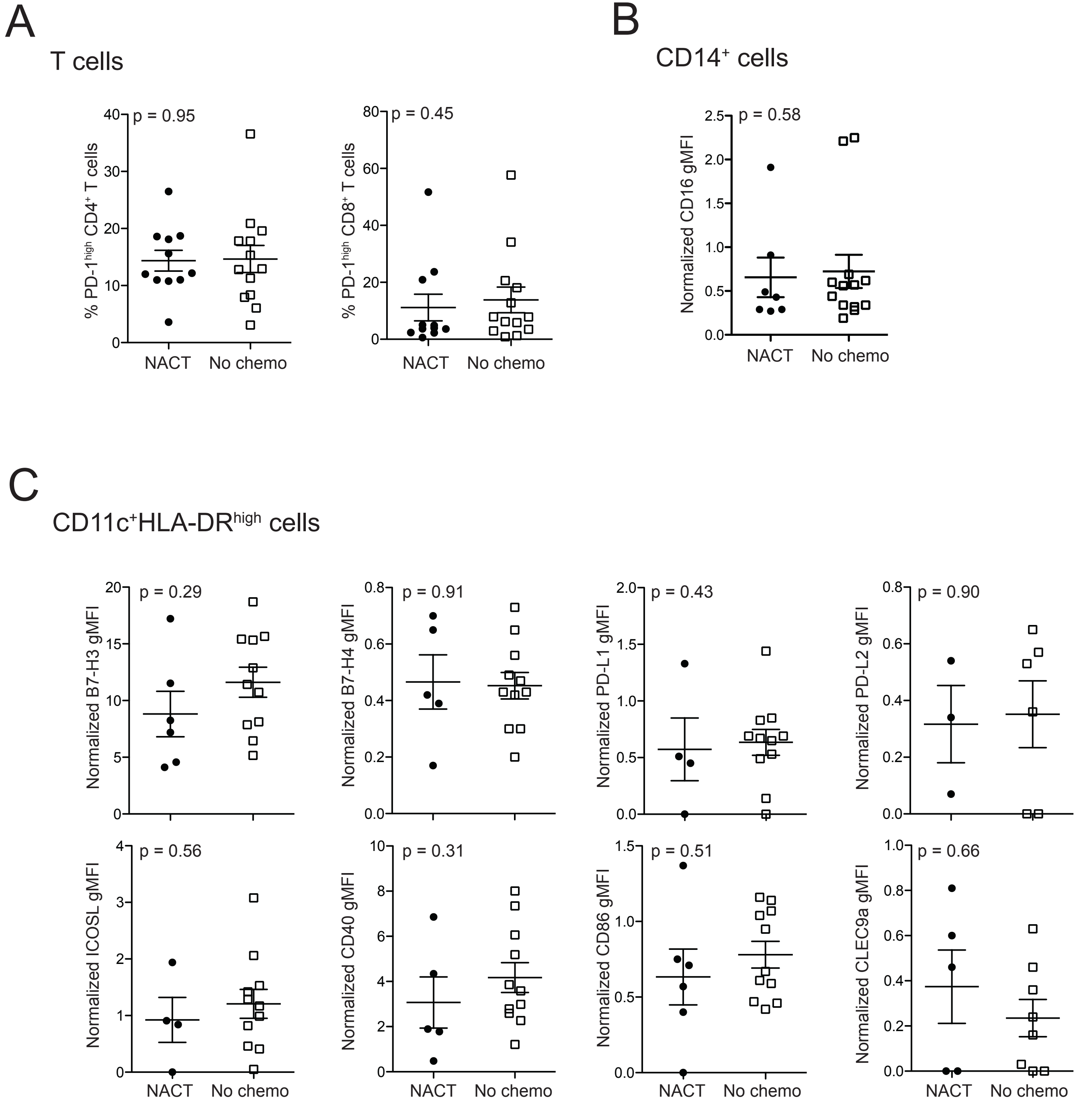
A
T cells
p = 0.95
% PD-1^high CD4+ T cells
p = 0.45
% PD-1^high CD8+ T cells
NACT
No chemo
B
CD14+ cells
p = 0.58
Normalized CD16 gMFI
C
CD11c+HLA-DR^high cells
p = 0.29
Normalized B7-H3 gMFI
p = 0.91
Normalized B7-H4 gMFI
p = 0.43
Normalized PD-L1 gMFI
p = 0.90
Normalized PD-L2 gMFI
p = 0.56
Normalized ICOSL gMFI
p = 0.31
Normalized CD40 gMFI
p = 0.51
Normalized CD86 gMFI
p = 0.66
Normalized CLEC9a gMFI

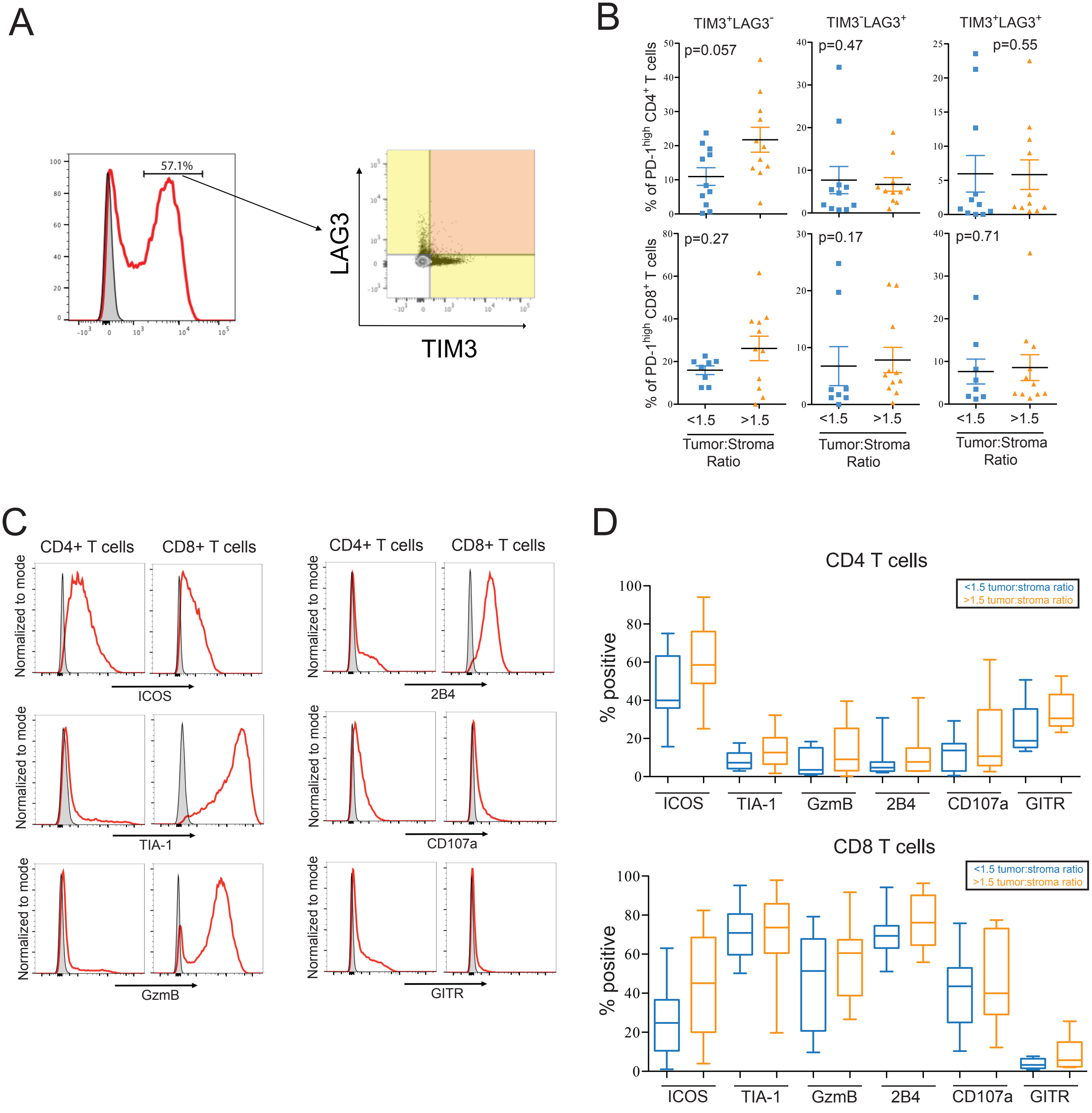
A
57.1%
LAG3
TIM3
B
TIM3+LAG3-
TIM3-LAG3+
TIM3+LAG3+
% of PD-1high CD4+ T cells
% of PD-1high CD8+ T cells
p=0.057
p=0.47
p=0.55
p=0.27
p=0.17
p=0.71
<1.5
>1.5
Tumor:Stroma Ratio
C
CD4+ T cells
CD8+ T cells
Normalized to mode
ICOS
TIA-1
GzmB
2B4
CD107a
GITR
D
CD4 T cells
CD8 T cells
% positive
<1.5 tumor:stroma ratio
>1.5 tumor:stroma ratio
ICOS
TIA-1
GzmB
2B4
CD107a
GITR

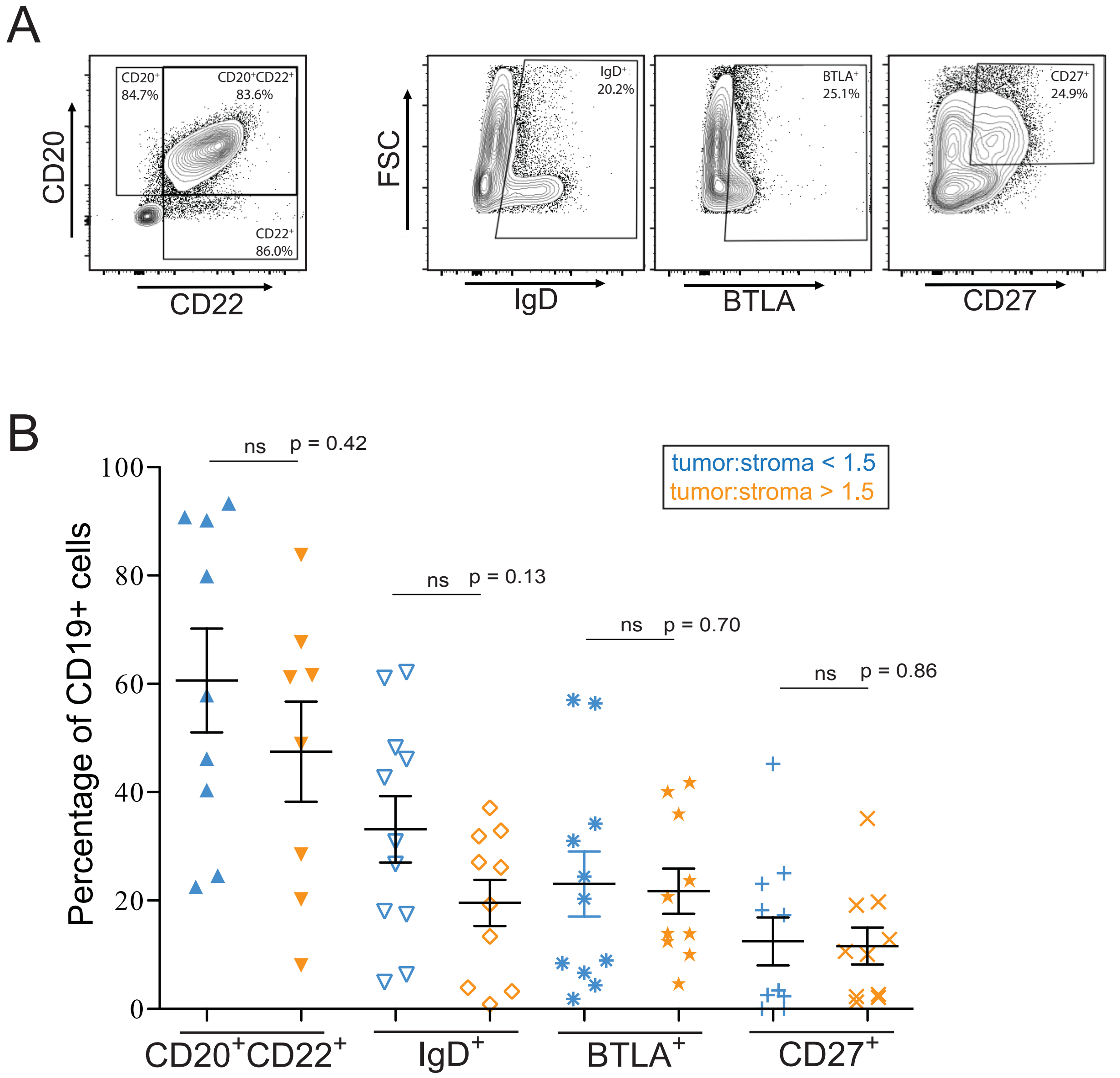

A
CD20+
84.7%
CD20+CD22+
83.6%
CD22+
86.0%
CD20
CD22
IgD+
20.2%
BTLA+
25.1%
CD27+
24.9%
FSC
IgD
BTLA
CD27
B
ns p = 0.42
ns p = 0.13
ns p = 0.70
ns p = 0.86
tumor:stroma < 1.5
tumor:stroma > 1.5
Percentage of CD19+ cells
100
80
60
40
20
0
CD20+CD22+
IgD+
BTLA+
CD27+

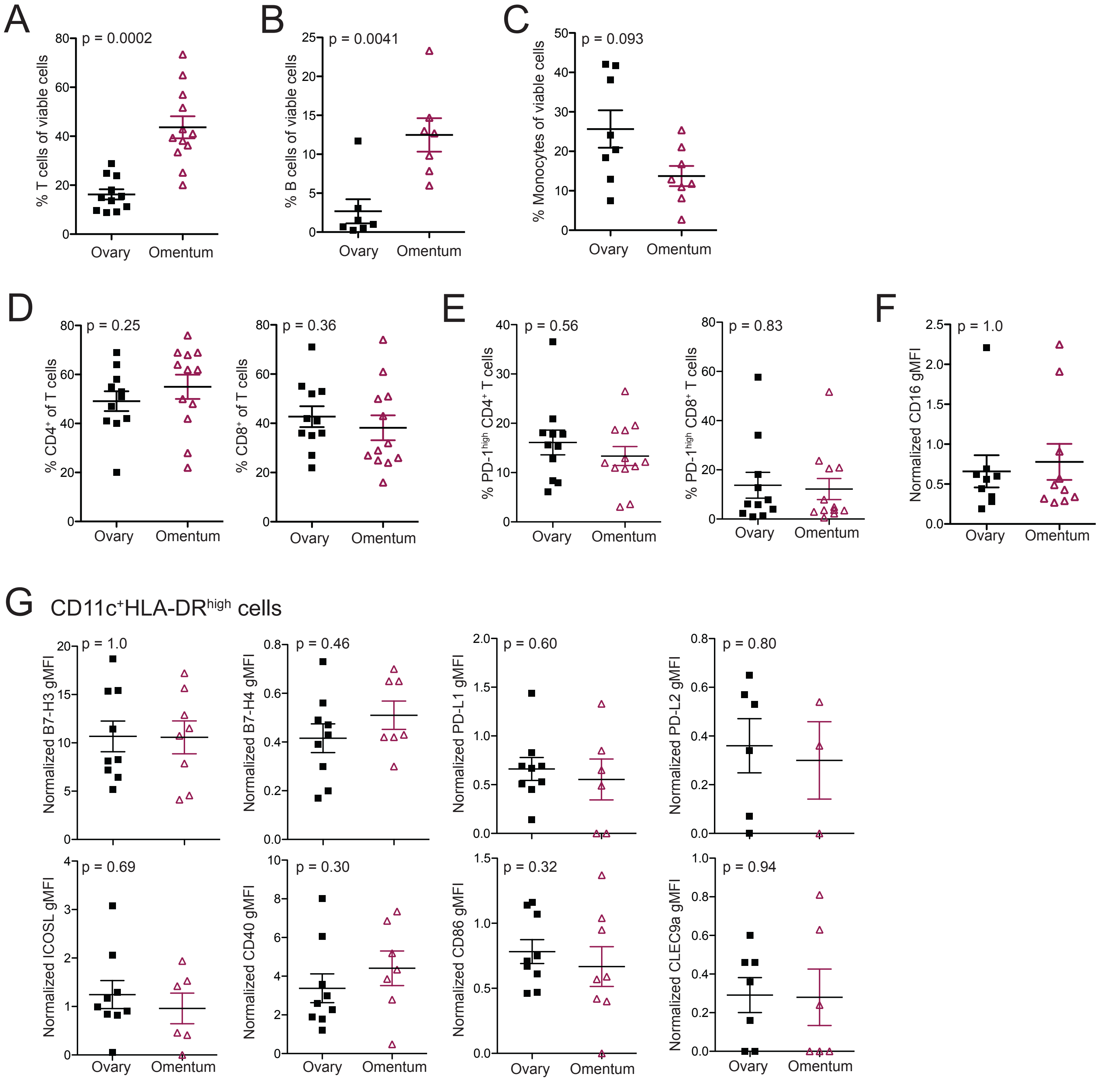
A
p = 0.0002
% T cells of viable cells
B
p = 0.0041
% B cells of viable cells
C
p = 0.093
% Monocytes of viable cells
D
p = 0.25
% CD4⁺ of T cells
p = 0.36
% CD8⁺ of T cells
E
p = 0.56
% PD-1high CD4⁺ T cells
p = 0.83
% PD-1high CD8⁺ T cells
F
p = 1.0
Normalized CD16 gMFI
G
CD11c⁺HLA-DRhigh cells
p = 1.0
Normalized B7-H3 gMFI
p = 0.46
Normalized B7-H4 gMFI
p = 0.60
Normalized PD-L1 gMFI
p = 0.80
Normalized PD-L2 gMFI
p = 0.69
Normalized ICOSL gMFI
p = 0.30
Normalized CD40 gMFI
p = 0.32
Normalized CD86 gMFI
p = 0.94
Normalized CLEC9a gMFI
Ovary
Omentum
